# Iron Oxide Nanozyme as Reactive Oxygen and Nitrogen Species Scavenger to Regulate Microglial Homeostasis in Stroke

**DOI:** 10.1002/advs.202518191

**Published:** 2026-01-20

**Authors:** Yilin Qi, Chunxiao Wang, Yuqing Miao, Jiamin Li, Zengyu Xun, Di Sun, Fei Xu, Minrui Liu, Heping Wang, Galong Li, Xuyi Chen, Haiming Fan, Xue Xue

**Affiliations:** ^1^ State Key Laboratory of Medicinal Chemical Biology College of Pharmacy Haihe Education Park Nankai University Tianjin China; ^2^ Key Laboratory of Synthetic and Natural Functional Molecule of the Ministry of Education College of Chemistry and Materials Science Northwest University Xi'an China; ^3^ Institute for Chinese Medicine Frontier Interdisciplinary Science and Technology Shaanxi University of Chinese Medicine Shaanxi China; ^4^ State Key Laboratory of Advanced Medical Materials and Devices Tianjin Institutes of Health Science Institute of Radiation Medicine Chinese Academy of Medical Sciences & Peking Union Medical College Tianjin China; ^5^ School of Biomedical Engineering Air Force Medical University Xi'an China; ^6^ Characteristic Medical Center of People's Armed Police Forces Tianjin China; ^7^ Academy for Advanced Interdisciplinary Studies Nankai University Tianjin China

**Keywords:** iron oxide nanozyme, ischemic stroke, microglia, neuroinflammation, RONS

## Abstract

In ischemic stroke, microglia adopt a pro–inflammatory M1‐like phenotype, which plays a pivotal role in the excessive production of nitric oxide radical (NO). The elevated levels of NO contribute to caspase‐mediated apoptosis, resulting in significant disruption of cerebral tissue architecture and consequent loss of brain function. In this study, we demonstrate that iron oxide nanoparticles (IONPs) with intrinsic enzyme–like activities can effectively scavenge NO by forming a nitrosyl‐metal complex. Specifically, 6 nm iron oxide nanoparticle (IONP6) exhibits enzyme‐like activities, superoxide dismutase (SOD) and catalase (CAT), thus potentially possessing the ability to scavenge the Reactive Oxygen and Nitrogen Species (RONS), especially the ability to scavenge NO. Furthermore, we show that IONP6 promotes the polarization of microglia toward the M2 phenotype, thereby alleviating neuroinflammation in both in vitro oxygen and glucose deprivation (OGD) and in vivo permanent middle cerebral artery occlusion (pMCAO) stroke models. This is achieved through the modulation of the HIF‐1α/TIM‐3 signaling axis in stroke rats. Additionally, IONP6 administration significantly reduces infarct size and improves neurological outcomes in stroke rats. Our findings position IONP6 as a promising drug‐free therapeutic agent for stroke, capable of regulating microglial polarization and mitigating secondary injury caused by the inflammatory cascade induced by NO.

## Graphical abstract

6 nm iron oxide nanoparticles (IONP6) exhibit enzyme‐like activities that scavenge reactive oxygen and nitrogen species (RONS), including nitric oxide (NO). In stroke models, IONP6 promotes microglial polarization toward the M2 phenotype, reduces neuroinflammation, and improves neurological outcomes, offering a promising drug‐free approach to mitigate stroke–induced brain injury and secondary damage.

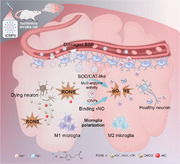

## 1 Introduction

Ischemic stroke, a life‐threatening arterial occlusion disorder, results in high morbidity and mortality worldwide, which is usually associated with acute neuroinflammation [1, 2]. Despite the proven benefit of early thrombolysis for ischemic stroke, strict eligibility criteria and a 4.5 h therapeutic window mean that many patients do not fully benefit, underscoring the urgent need for adjunctive therapies [3]. At present, sterile inflammation initiated after cerebral ischemia is considered a potential therapeutic target to expand the therapeutic timeframe [4]. Microglia, resident immune cells in the brain, sense changes in the brain environment and polarize into different subsets, including pro‐inflammatory M1 and wound‐healing M2 [5]. Pro‐inflammatory M1 microglia promote excessive production of reactive oxygen species (ROS) and reactive nitrogen species (RNS), leading to neurodegeneration and neuronal death [6, 7]. ROS include species, such as the hydroxyl radical (•OH), superoxide (•O_2_^−^), and hydrogen peroxide (H_2_O_2_). RNS include nitric oxide (NO), and its derivative, the peroxynitrite (ONOO^−^), a powerful oxidant, able to damage many biological molecules [8, 9]. During ischemic stroke, excessive NO production from endothelial cells, neurons, and activated microglia exacerbates tissue damage by promoting neuroinflammation and oxidative stress. The imbalance of NO production leads to a cascade of RNS formation, which includes ONOO^−^, a highly reactive molecule that can damage cellular components like lipids, proteins, and DNA, leading to neuronal injury and death. The major toxic effect for generation of NO by pro‐inflammatory M1 microglia is harmful to proteins, membrane lipids, and DNA, dramatically aggravating oxidative stress in ischemic stroke [10]. ONOO^−^ has also been implicated as a causative factor in the disruption of the blood‐brain barrier (BBB) in some neurologic diseases, owing to its much higher penetrating capacity across lipid bilayers [11]. Therefore, dampening the toxic effects of post‐stroke inflammation by scavenging ROS and RNS (RONS), especially NO, will be greatly important to develop methods for the treatment of stroke [12].

Small molecule drugs such as nitrite/nitrate compounds with the effect of scavenging NO have poor activity and cannot be used in clinical applications [13, 14]. In recent years, due to the unique properties of simple preparation, high stability, and adjustable activity, nanomaterials have attracted extensive attention and are widely used in the biomedical field. Thus far, exciting paradigms can be found in the NO clearance, such as metal‐based nanomaterials [14, 15, 16], organic nanomaterials [17], etc. In these preclinical experiments, the effectiveness and advantages of nanotherapy for scavenging NO in stroke have been clearly proven. Nevertheless, for the currently developed nanomaterials, complicated preparation methods, difficult large‐scale production, and limited biocompatibility are enormous challenges for clinical translation. In addition, the toxicity of these nanomaterials is still unclear due to the lack of in vivo distribution and metabolic data. Therefore, it is very worthwhile to develop a safer and more effective NO scavenger to supply neuroprotection and relieve the neurological damage after ischemic stroke. Iron oxide nanoparticles (IONPs, including Fe_3_O_4_ and Fe_2_O_3_), which present excellent biocompatibility, are inorganic functional nanomaterials approved by the FDA for clinical application [18]. Due to their unique properties, like magnetic activity, IONPs have received increasing attention as magnetic resonance imaging [19, 20], drug carriers [21], and thermotherapy nanomaterials in biomedical applications [22, 23]. Previous studies have shown that IONPs have superoxide dismutase‐like (SOD‐like) and catalase‐like (CAT‐like) activity, which can degrade ROS (H_2_O_2_) to produce O_2_ [24, 25]. These enzyme‐like properties indicate that IONPs are a type of nanomaterial‐based enzyme mimics, known as iron oxide nanozymes [26, 27]. More specifically, the enzyme‐like activity of IONPs exerts neuroprotective effects by not only scavenging harmful ROS but also converting them into O_2_, which is urgently required by neurons in ischemic stroke. This dual action provides neuroprotection and offers the possibility of extending the therapeutic window for stroke treatment. However, the research on the removal of RNS (NO, ONOO^−^) by IONPs is insufficient. Inspired by the rapid reaction between hemoglobin and NO to form HbFe(II)NO [28], we speculate that ferrous iron in IONPs can also react with NO, which can be used to remove NO. However, high content of ferrous iron leads to oxidative stress [29]. The ferrous iron content is related to the particle size. The fraction of Fe(II) increased as particle size increased [30]. So, we believe that IONPs with an appropriate particle size can be used as an NO scavenger. Above all, we hypothesized that IONPs have the potential to scavenge NO through the reaction of ferrous iron with NO, so that they can treat inflammation‐related diseases, such as ischemic stroke.

**SCHEME 1:**
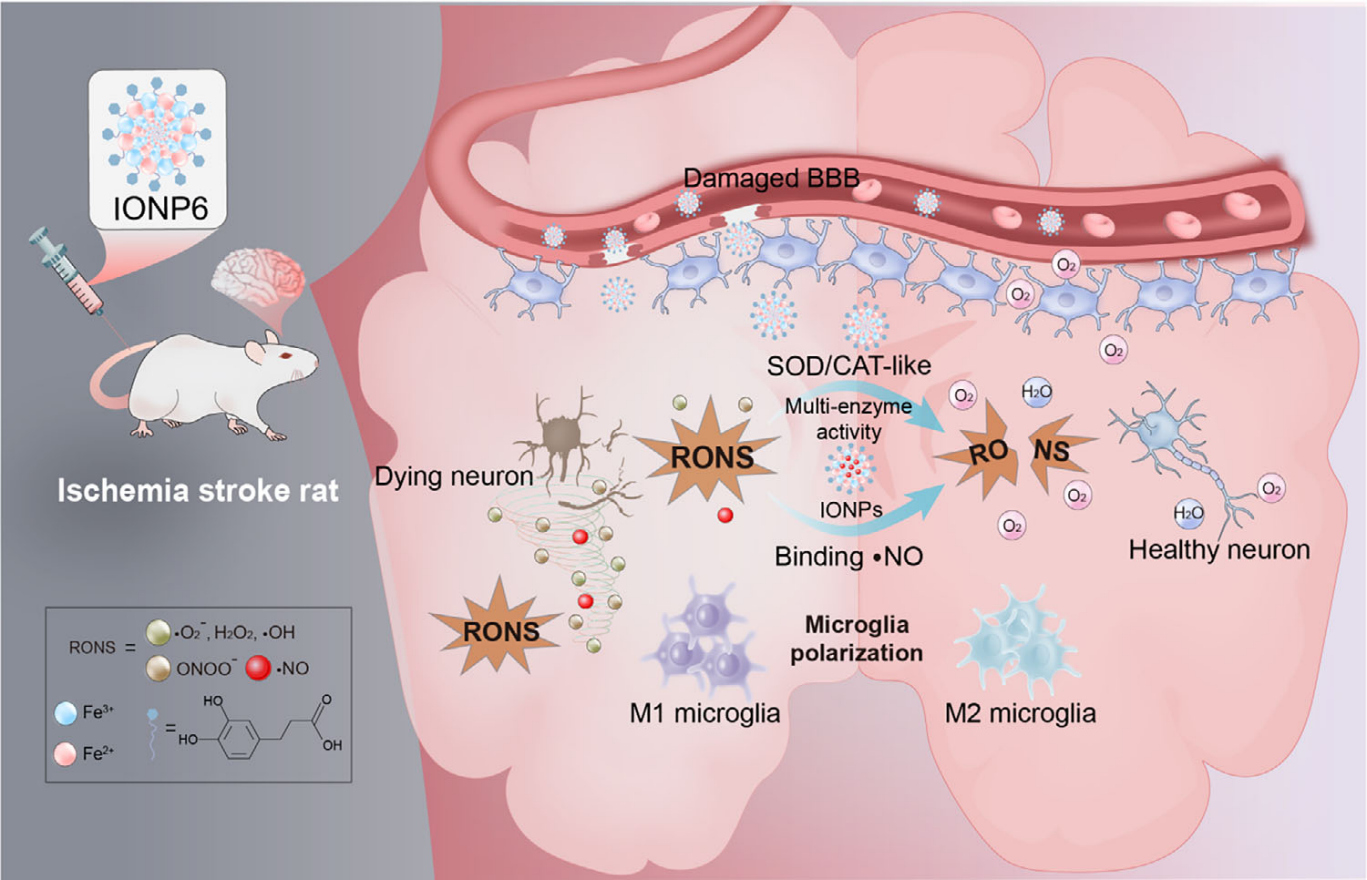
Schematic illustration of microglia polarization modulation by IONP6 for the treatment of cerebral ischemia to scavenge RONS and attenuate the effects of inflammatory injury on neurons in vivo. Cerebral ischemia produces RONS to trigger a pro‐inflammatory response, driving microglia polarization toward the M1 phenotype. The activated M1 microglia release NO and other inflammatory mediators, promoting neuronal apoptosis. Upon intravenous administration, IONP6 effectively crosses the blood‐brain barrier and scavenges RONS to modulate the inflammatory microenvironment. Specifically, IONP6 scavenges RONS, especially NO, and drives the polarization of microglia to the M2 phenotype. The alleviation of neuroinflammation contributes to the recovery of neurological function in stroke rats.

Here, we described the evolution of nanozymes IONPs for stroke treatment. Our findings revealed that 3,4‐dihydroxyhydrocinnamic (DHCA), which exhibited strong antioxidant properties and neuroprotective effects, modified IONP6 exhibited enzyme‐like activities and reacted rapidly with NO to form metal‐nitrosyl complexes. IONP6 displayed extensive antioxidative activities against multiple toxic RNS, including ONOO^−^ and NO, emphasizing their potential as a robust RNS scavenger. In vitro results further proved that IONP6 had anti‐inflammatory activity by scavenging RONS and promoting the transformation of microglia to the M2 phenotype. Owing to the realization of anti‐inflammatory effect, IONPs effectively inhibited the inflammatory response and reconstituted the brain microenvironment in a widely used stroke rat model of permanent middle cerebral artery occlusion (pMCAO), which was accompanied by excessive production of RONS and overactivated microglia, promoting the neuronal recovery and regeneration. In this study, it is an attractive strategy to eliminate RONS, especially NO, by using the enzyme‐like activity of IONP6 to inhibit inflammation, to achieve the goal of treating stroke (Scheme 1).

## 2 Results and Discussion

### 2.1 Synthesis and Characterization of IONPs

**FIGURE 1:**
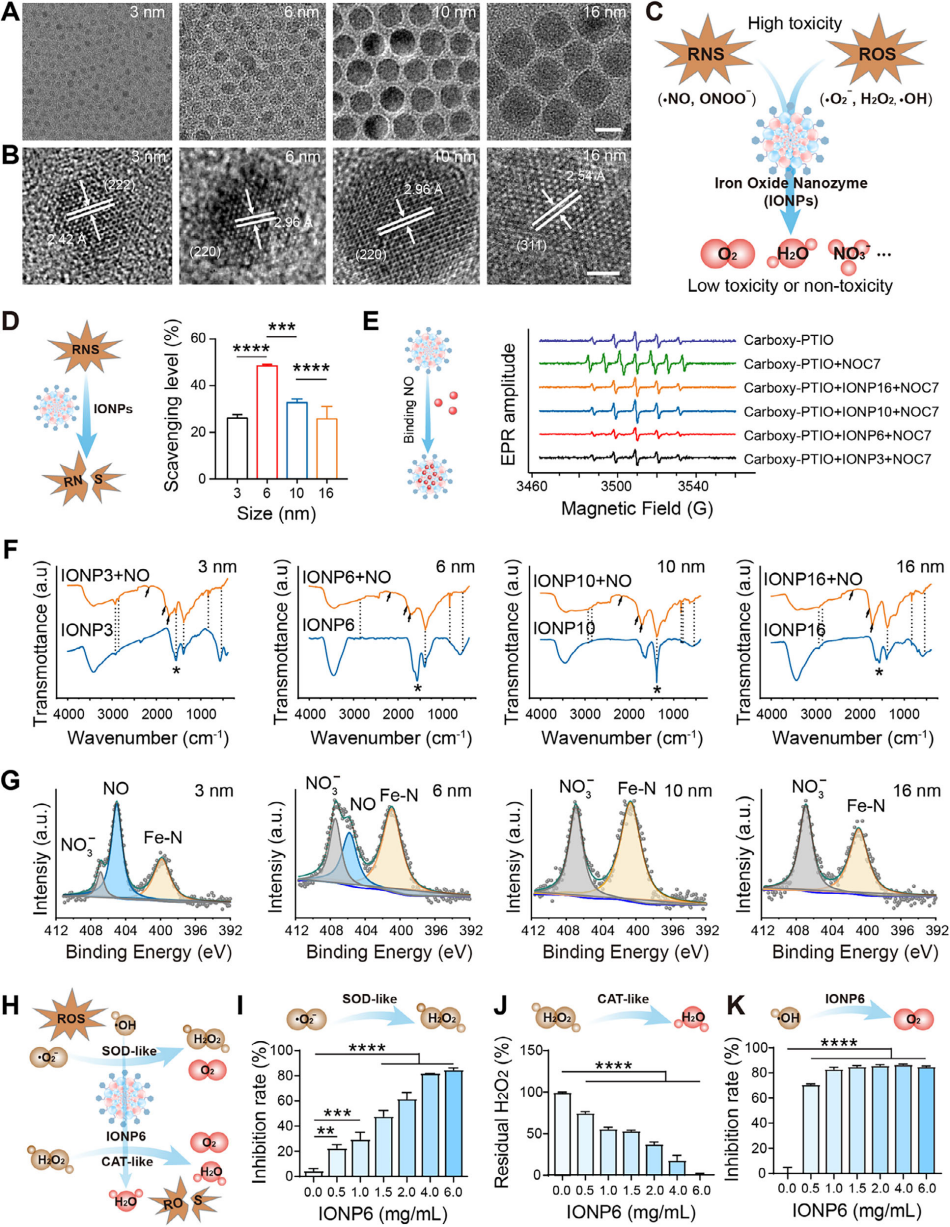
Characterization of iron oxide nanoparticles (IONPs) and their scavenging effects on reactive oxygen and nitrogen species (RONS). (A) Transmission electron microscope images of 3 nm IONPs (IONP3), 6 nm IONPs (IONP6), 10 nm IONPs (IONP10), and 16 nm IONPs (IONP16). Scale bar, 10 nm. (B) High‐resolution transmission electron microscope images of IONPs. Scale bar, 2 nm. (C) Illustration of RONS scavenging by IONPs. (D) RNS scavenging activities of the IONPs were detected by DPPH assay. IONP6 displayed superior RNS scavenging activity (n = 3 independent experiments). (E) Schematic diagram of the mechanism for scavenging NO (left) and NO scavenging activities of IONP3, IONP6, IONP10, and IONP16 were detected by electron paramagnetic resonance (EPR) (right). The NO scavenging mechanism was that Fe^2+^ could react fast with NO to form stable nitrosyl‐metal complexes. (F) Fourier transform infrared spectroscopy of IONP3, IONP6, IONP10, and IONP16 (blue line) and reaction products of IONPs and NO (orange line). (G) XPS analysis of reaction products of IONP and NO, showing the binding energy (BE) levels of N1s. (H to K) The SOD‐like, CAT‐like activity and the •OH inhibition rate of IONP6. The schematic mechanism of SOD‐like, CAT‐like activity and the •OH scavenging of IONP6 (H). The SOD‐like (I), CAT‐like (J) activity and the •OH inhibition rate of IONP6 (K) (n = 3 independent experiments). The data are presented as the mean ± SEM. *p*‐values are calculated by one‐way analysis of variance (ANOVA), ^***^ *p*< 0.001, ^****^ *p*< 0.0001, IONP3, IONP10 or IONP13 vs. the IONP6 group; ^**^ *p*< 0.01, ^***^ *p*< 0.001, ^****^ *p*< 0.0001, 0.5, 1.0, 1.5, 2.0, 4.0 or 6.0 mg/mL IONP6 vs. the 0.0 mg/mL IONP6 group.

IONPs were synthesized using the well‐established heat‐up method according to previous reports [31]. The core size and morphology of the synthesized IONPs were investigated using transmission electron microscopy (TEM). As shown by TEM images (Figure 1A), the distribution of IONPs was uniform and nearly spherical with diameters of 3, 6, 10, and 16 nm, respectively. High‐resolution TEM (HRTEM) images of IONPs exhibited distinct lattice fringe patterns, indicating the single‐crystalline nature of the nanocrystals (Figure 1B). DHCA exhibited strong antioxidant properties and neuroprotective effects [15, 23, 32], which led to its use in modifying IONPs. The subsequent ligand (DHCA) exchange resulted in the formation of high‐quality, hydrophilic IONPs. The hydrodynamic size of the four DHCA‐modified IONPs was increased to 8.6, 18, 20, and 28.4 nm (Figure S1A), due to the presence of hydrated layers based on dynamic light scattering measurements, respectively. Figure S1B showed the X‐ray diffraction (XRD) patterns of the synthesized samples. All diffraction peaks could be exclusively indexed as cubic spinel iron oxide (JCPDS card number 19–0629), which was identified by the (220), (311), (400), (422), (511), and (400) reflections. Fourier transform infrared spectroscopy (FT‐IR) data (Figure S2 and Table S1) indicated the presence of carboxylate bands (1561 cm^−1^, marked with ^*^) arising from DHCA on the surface of IONPs. These results are consistent with a previous report on the synthesis of hydrophilic DHCA‐modified IONPs, indicating the successful synthesis of our IONPs [33].

### 2.2 Dual Enzyme‐Like Activities and RONS Scavenging Activities of the IONPs

In the acute phase of stroke, the elevated levels of RONS lead to the infiltration of peripheral immune cells, exacerbating neuronal loss [34]. Therefore, scavenging RONS, especially NO, may represent an effective strategy for the treatment of ischemic stroke [35]. We initially employed the classic DPPH• (2,2‐di‐(4‐tert‐octylphenyl)‐1‐picrylhydrazyl radical) assays to assess the capacity of IONPs to scavenge RNS (Figure 1C). The DPPH• assay, a widely recognized method for assessing free radical scavenging activity, was employed to evaluate the free radical scavenging potential of the nanomaterials. Figure 1D revealed that all types of IONPs could effectively eliminate DPPH•. Nevertheless, IONP6 displayed superior DPPH• scavenging activity as compared to IONP3, IONP10, and IONP16. The inhibitory rate of DPPH• by IONP6 reached up to 48%, which was 1.9 times, 1.5 times, and 2 times higher than IONP3, IONP10, and IONP16, respectively. Moreover, we investigated the effects of IONPs with different particle sizes on NO using electron paramagnetic resonance (EPR) spectroscopy, with carboxy‐PTIO as the indicator and NOC7 as the NO donor. Carboxy‐PTIO was used to form a stable carboxy‐PTI complex with NO, resulting in an increase in the EPR signal from 5 to 7 lines [17]. Upon the addition of IONPs, the EPR data revealed that IONP6 exhibited the highest NO scavenging activity among these four types of IONPs, which was attributed to its superior specific surface area and ferrous iron content (Figure 1E). Although the ferrous iron content decreases with smaller particle sizes, the specific surface area increases [30]. It explained the poorer NO scavenging effects of IONP3, IONP10, and IONP16, compared with the IONP6. The EPR signal of carboxyl‐PTI produced by carboxyl‐PTIO and NO completely disappeared after incubation with IONP6, which indicated the complete removal of NO and even more stable carboxyl‐PTIO.

Additionally, we explore the mechanism of IONPs on NO scavenging. As the new bands of N‐O around 1712 cm^−1^ and Fe‐N around 2168 cm^−1^ were shown, we initially demonstrated that Fe^2+^ could react fast with NO to form stable nitrosyl‐metal complexes (Figure 1F; Table S1). We further evaluated the binding ability of IONPs with NO using XPS and analyzed the reaction products. Figure 1G showed XPS spectra of the N1s. The products of the reaction between IONPs and NO could be deconvoluted into three peaks with binding energies of 399.8, 405.0, and 406.9 eV for IONP3, while the peaks at 400.3, 405.2, and 406.8 eV for IONP6 were assigned to Fe‐N, NO, and NO_3_^−^, respectively. For IONP10 and IONP16, the XPS spectra of N1s could be deconvoluted into two peaks. The binding energy at 400.0 eV for the IONP10 and 400.1 eV for the IONP16 is attributed to Fe─N. The peaks at 406.3 eV for the IONP10 and 406.2 eV for the IONP16 were assigned to NO_3_^−^ (Table S2). In addition to the Fe─N peak representing the nitrosyl‐metal complexes, the NO peak may be caused by the adsorption of NO on the surface of IONPs, and only exists in IONPs with a larger specific surface area of IONP3 and IONP6. This demonstrates that the superior NO scavenging activity of IONP6 is attributed to the adsorption of NO by the modifying group, which facilitates the reaction between NO and Fe^2+^ in IONP6. This also highlights the importance of specific surface area in the NO scavenging by IONPs. However, the NO_3_^−^ peak may result from the reaction of NO with O_2_. In conclusion, our results demonstrate that IONP6 with dual enzyme‐like activity effectively scavenges RONS, especially NO. The high efficiency of NO elimination is primarily attributed to the active iron centers of IONPs, which is consistent with various metal‐based catalytic reactions.

We next evaluated the enzyme‐like activity of IONP6 and its capacity to scavenge ROS through enzymatic assays (Figure 1H). The SOD‐like activity of IONP6 was tested by suppressing the photoreduction of nitro blue tetrazolium (NBT). IONP6 exhibited SOD‐like activity, with a specific activity of 22 U/mg (Figure S3). Its ability to scavenge •O_2_^−^ increased in a concentration‐dependent manner (Figure 1I). The O2 generated from the decomposition of •O_2_^−^ also contributes to stroke recovery. To verify the CAT‐like activity, the residual H_2_O_2_ was calculated by monitoring the absorbance of H_2_O_2_ at 240 nm. As the concentration of IONP6 increased, the reaction proceeded faster, and the residual H_2_O_2_ decreased accordingly, further indicating pronounced CAT‐like activity (Figure 1J). Furthermore, •OH scavenging was evaluated using 3,3’,5,5’‐tetramethylbenzidine (TMB) as a chromogenic probe. •OH generated by the Fenton reaction oxidizes TMB to a blue product with a characteristic absorbance at 652 nm. Treatment with IONP6 markedly decreased the 652 nm signal, indicating potent •OH‐scavenging activity (Figure 1K). Collectively, IONP6 has dual antioxidant enzyme‐like activities and is capable of eliminating diverse RONS; consequently, it shows significant therapeutic potential against RONS‐driven neuroinflammation after stroke.

### 2.3 IONPs as RONS Scavengers to Suppress Neuroinflammation in Microglia

**FIGURE 2:**
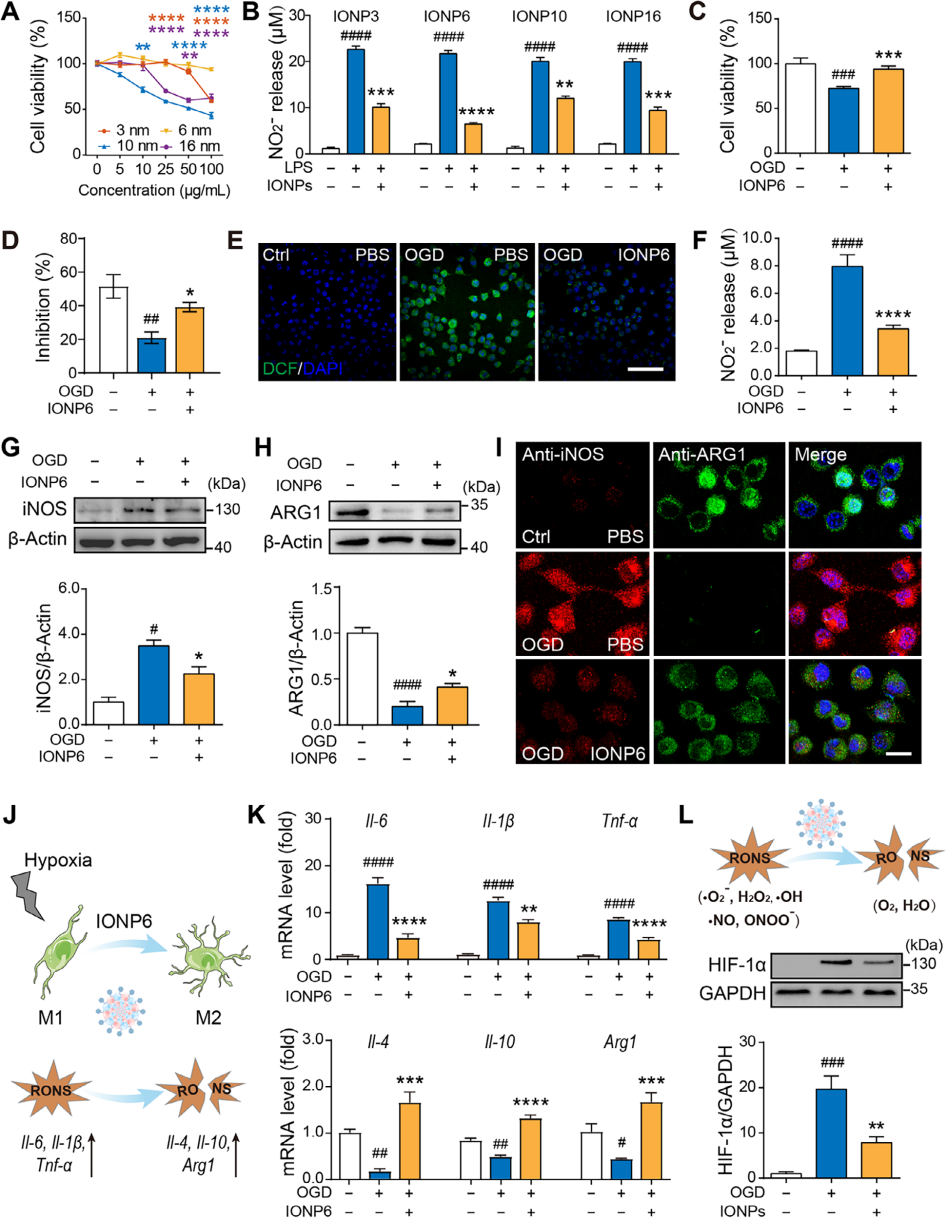
IONP6 cleared RONS in oxygen‐glucose deprivation (OGD)‐induced microglia and polarized microglia to the M2 phenotype. (A) Viabilities of BV‐2 cells treated with IONP3, IONP6, IONP10, and IONP16 for 24 h. Cells cultured without IONPs served as the control group. Cell viabilities were measured by the 3‐(4,5‐dimethylthiazol‐2‐yl)‐2,5‐diphenyltetrazolium bromide (MTT) assay and were shown as the percentage of untreated cells (n = 3–4 independent experiments). (B) Measurement of NO generation by Griess assay (n = 5 independent experiments). BV‐2 cells incubated with fresh medium without LPS induction served as the control group, cells induced with 0.1 µg/mL LPS for 24 h served as the LPS group, and cells induced with 0.1 µg/mL LPS and treated with IONPs for 24 h served as the IONPs group. (C) Mitigation of OGD‐induced cytotoxicity by 25 µg/mL IONP6 (n = 8 technical replicates from 3 independent experiments). OGD‐induced BV‐2 cells were treated with IONP6 or PBS for 6 h. (D) O2^−^ scavenging activities of IONP6 in OGD‐induced BV‐2 cells (n = 3 independent experiments). (E) Representative fluorescent images of cellular ROS levels with or without IONP6 treatment, stained by DCFH‐DA probe (n = 5 technical replicates from 3 independent experiments). Scale bar, 100 µm. (F) NO levels in OGD‐induced BV‐2 cells with or without treatment with 25 µg/mL IONP6 (n = 6 technical replicates from 3 independent experiments). (G and H) Western blot analysis of iNOS (G, n = 3 independent experiments) and ARG1 (H, n = 4 independent experiments) protein expression in OGD‐induced BV‐2 cells, with or without treatment with 25 µg/mL IONP6. OGD‐induced BV‐2 cells were treated with IONP6 or PBS for 6 h. (I) Immunofluorescent staining against iNOS (red), ARG1 (green), and nuclei (blue) of BV‐2 cells (n = 3 independent experiments). OGD‐induced BV‐2 cells were treated with 25 µg/mL IONP6 or PBS for 6 h. Scale bar, 25 µm. (J) Schematic diagram depicting the transformation of M1 phenotype microglia to M2 phenotype microglia induced by OGD and modulated by IONP6 treatment. (K) The mRNA expression levels of M1‐ (top) and M2‐related genes (bottom) were detected by RT‐qPCR in BV‐2 cells (n = 3 independent experiments). (L) Western blot analysis of Hif‐1α protein expression in OGD‐induced BV‐2 cells, with or without treatment with 25 µg/mL IONP6 (n = 3 independent experiments). The data are presented as the mean ± SEM. P‐values are calculated by one‐way ANOVA. BV‐2 cells incubated with fresh medium without OGD served as the control group. BV‐2 cells with OGD induced served as the OGD group. BV‐2 cells with OGD induced and treated with IONP6 served as the IONP6 group. ^###^ *p*< 0.001, ^####^ *p*< 0.0001, LPS group vs the control group, ^**^ *p*< 0.01, ^***^ *p*< 0.001, ^****^ *p*< 0.0001, IONPs group vs. the LPS group. ^#^ *p*< 0.05, ^##^ *p*< 0.01, ^###^ *p*< 0.001, ^####^ *p*< 0.0001, OGD group vs. the control group, ^*^ *p*< 0.05, ^**^ *p*< 0.01, ^***^ *p*< 0.001, ^****^ *p*< 0.0001, IONP6 group vs. the OGD group.

NO produces multiple regulatory effects in the inflammatory phase of a variety of nervous system diseases [35]. Especially, the concentration and signaling of NO change dramatically in the ischemic brain [34]. To investigate the NO scavenging effects directly on ischemic stroke, we chose an in vitro oxygen and glucose deprivation (OGD) model to study ischemia‐induced neuroinflammation in microglia. Initially, we assessed the potential toxicity of a series of IONPs in BV‐2 microglial cell lines. After incubation with varying concentrations of IONPs, a reduction in metabolic activity was observed in the larger‐sized IONPs (IONP10 and IONP16) (Figure 2A), while no significant cytotoxicity was detected for IONP6 at concentrations ≤100 µg/mL. In contrast, as a smaller particle size, IONP3 showed similar cell viability in a range of relatively low concentrations but greater toxicity at high concentrations (∼100 µg/mL), compared with IONP6. Our data showed that IONP6 has better biocompatibility and lower toxicity than that of other sizes of IONPs in microglia. The inhibitory effects of IONPs on NO overproduction were investigated in lipopolysaccharide (LPS)‐induced neuroinflammation in BV‐2 cells by Griess reagent [36]. Among them, IONP6 had the most obvious scavenging effect on RNS (NO) at the concentration of 25 µg/mL (Figure 2B). Subsequently, we conducted an in vitro OGD model to further confirm whether IONP6 eliminates RONS induced by OGD. IONP6 prevented OGD‐induced cell death (Figure 2C). The nitroblue tetrazolium (NBT) assay was employed to detect intracellular ROS levels and SOD enzyme activity following treatment with IONPs. With the significant increase in ROS inhibition efficiency by IONP6 (Figure 2D), the intracellular SOD enzyme activity in BV‐2 cells treated with IONP6 also significantly increased (Figure S4A). We used DCFH‐DA, a green fluorescence probe, to prove changes in intracellular ROS levels. OGD‐induced BV‐2 cells with ROS generation accelerated cellular damage, which was confirmed by the appearance of bright green fluorescence. The fluorescence signal was significantly reduced, indicating that IONP6 effectively scavenges reactive oxygen species in vitro (Figure 2E; Figure S4B). Furthermore, IONP6 dramatically attenuated the NO release in OGD‐induced BV‐2 cells (Figure 2F). All results indicate that IONP6 consistently outperforms other types of IONPs and exhibits the ability to scavenge RONS in vitro.

### 2.4 Effects of IONPs on Promoting Microglial M1 to M2 Polarization in the OGD Model

Previous studies have reported that neuroinflammation is dynamically regulated by microglia polarization in the presence of OGD [37]. The above data proved that IONP6 inhibited neuroinflammation, which is closely related to microglia subsets. The M1 phenotype is linked to a pro‐inflammatory response, whereas the M2 phenotype is associated with anti‐inflammatory responses and tissue repair. M1 microglia have been reported to release various pro‐inflammatory factors, such as interleukin‐6 (IL‐6), interleukin‐1 beta (IL‐1β), etc., which promote inflammation, cause neural damage, and retard neurogenesis. In contrast, M2 microglia play a key role in resolving inflammation and promoting neuroprotection. The anti‐inflammatory and neurotrophic factors, such as interleukin‐10 (IL‐10) and interleukin‐4 (IL‐4), are produced by M2 microglia [38]. The activation of the M2 phenotype is typically driven by anti‐inflammatory cytokines like IL‐10 and IL‐4. The transition to the M2 phenotype facilitates the repair of damaged brain tissue, clears debris, and supports neurogenesis. Therefore, we next investigate whether IONP6 can regulate microglia polarization. The expression levels of inducible nitric oxide synthase (iNOS, M1 microglia maker) and Arginase1 (ARG1, M2 microglia maker) were determined in BV‐2 cells. Additionally, activation of iNOS directly contributes to the excessive production of ROS, with iNOS expression levels serving as an indicator of ROS levels. The expression of iNOS was most prominent in OGD‐induced BV‐2 cells, but treatment with IONP6 significantly reduced the OGD‐induced iNOS expression in these cells (Figure 2G). In contrast to the change in iNOS expression, IONP6 treatment enhanced ARG1 expression (Figure 2H). These results were further delineated by flow cytometry analysis. After IONP6 treatment, the number of iNOS‐positive cells decreased to ∼35% compared with the OGD group, and the number of ARG1‐positive cells increased to ∼97% (Figure S5A), suggesting a successful transformation of microglia from M1 to M2 phenotype. In our study, IONP6 inhibited the inflammation by scavenging RONS, especially NO. Benefiting from the changes in the inflammatory environment, IONP6 promotes the transformation of microglia to the M2 phenotype.

Immunofluorescence staining of iNOS and ARG1 revealed that IONP6 decreased the number of iNOS‐positive cells and increased the number of ARG1‐positive cells in OGD‐induced BV‐2 cells (Figure 2I; Figure S5B), which was consistent with the above conclusion. Hence, we analyzed typical inflammatory factors and anti‐inflammatory factors under OGD conditions with or without IONP6 treatment. Quantitative polymerase chain reaction (RT‐qPCR) analysis showed that IONP6 significantly downregulated the expressions of pro‐inflammatory mRNA such as *Il‐6*, tumor necrosis factor‐α (*Tnf‐α*), and *Il‐1β*, while the expression of *Il‐4*, *Il‐10*, and *Arg1* showed an increasing trend (Figure 2J,K). Hypoxia‐inducible factor 1 alpha (HIF‐1α) is associated with ROS in hypoxic conditions in the brain and is linked to the exaggerated activation of microglia. Therefore, we evaluated the expression of HIF‐1α to verify whether the O_2_ produced after RONS scavenging by IONP6 improved the hypoxic microenvironment. The results showed that HIF‐1α expression was significantly increased in OGD‐induced BV‐2 cells, while HIF‐1α expression was markedly reduced in cells treated with IONP6 (Figure 2L). The data described above indicate that IONP6 suppresses the neuroinflammatory response in the OGD model by effectively scavenging RONS, especially NO, and promoting the transformation of microglia from the M1 phenotype to the M2 phenotype.

### 2.5 The Biodistribution of IONP6 in the Stroke Rat Model of pMCAO

**FIGURE 3:**
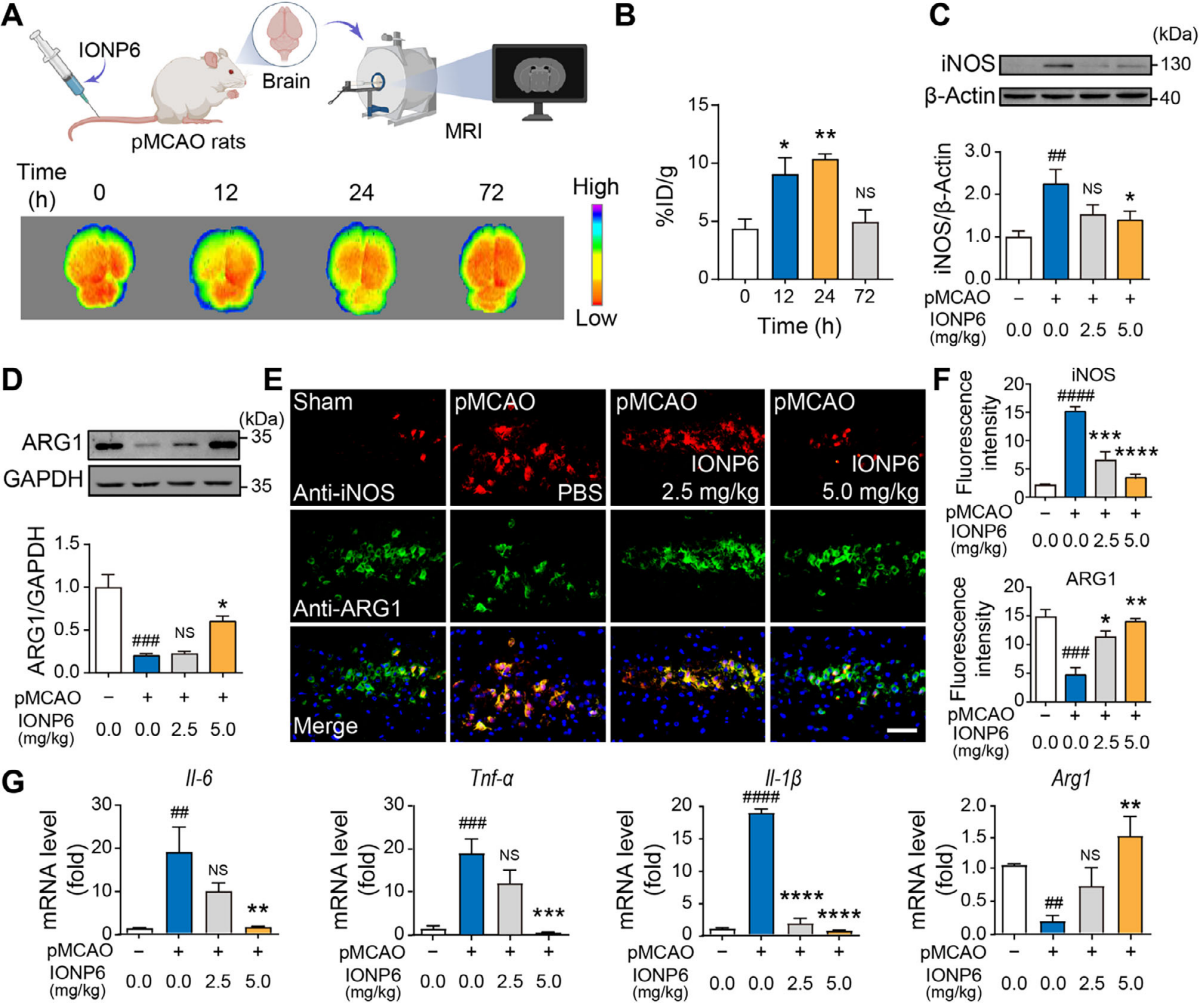
The distribution and anti‐neuroinflammatory effects of IONP6 in vivo in the stroke rat model (pMCAO rats). (A) Schematic diagram and MRI images of IONP6 penetrating the BBB. IONP6 accumulated in the brain mainly through the BBB leakage gap caused by stroke. The color scale in the figure indicates the relative concentration of IONP6, with higher intensity (represented by blue) corresponding to greater iron accumulation in the brain and lower intensity (represented by red/yellow) indicating lower accumulation. (B) Efficiency of IONP6 distribution in the brains at different time points. The brains of pMCAO rats (n = 3–4 rats per time point), intravenously injected with 5 mg/kg IONP6, were collected at the indicated times and analyzed using an Inductively Coupled Plasma Optical Emission Spectrometer (ICP‐OES). The concentration of IONP6 was normalized as the percentage of the injected dose (ID) per gram of each organ (% ID/g). (C) Western blot analysis of iNOS protein. The corresponding statistical graph below quantifies the iNOS/β‐Actin ratio across the treatment groups. (n = 3 rats per group). (D) Western blot analysis of ARG1 protein. The corresponding statistical graph below quantifies the ARG1/GAPDH ratio across the treatment groups. (n = 3 rats per group). (E) Immunohistochemistry analysis of ARG1 (green) and iNOS (red) in brains from sham rats, pMCAO rats with or without IONP6 treatment. Scale bar, 12.5 µm. (F) Quantitative analysis of different groups in panel E (n = 3 rats per group). (G) The mRNA expression levels of *Il‐6*, *Tnf‐α*, *Il‐1β* and *Arg1* were detected by RT‐qPCR in the infarction region of the ipsilateral cerebral hemisphere from sham rats, pMCAO rats with or without IONP6 treatment (n = 3 rats per group). (C–G) proved that IONP6 inhibited neuroinflammation in the stroke rat model. The data are presented as the mean ± SEM. P‐values are calculated by one‐way ANOVA. Rats without IONP6 treatment served as the sham group, pMCAO rats without IONP6 treatment served as the pMCAO group, and pMCAO rats with IONP6 treatment served as the IONP6 group. ^*^ *p* < 0.05, ^**^ *p* < 0.01, 12 or 24 h group vs. 0 h group, no statistical significance (NS), 72 h group vs. 0 h group, ^##^ *p* < 0.01, ^###^ *p* < 0.001, ^####^ *p* < 0.0001, pMCAO group vs. the sham group, NS, ^*^ *p* < 0.05, ^**^ *p* < 0.01, ^***^ *p* < 0.001, ^****^ *p* < 0.0001, 2.5 or 5 mg/kg IONP6 group vs. the pMCAO group.

To explore the in vivo biodistribution of IONP6, we next demonstrated the biodistribution in pMCAO rats after intravenous injection of IONP6. The stroke rats were intravenously injected with 5 mg/kg IONP6 at different times, and the content of iron ions in different organs was quantified. The results suggested that IONP6 has a rapid initial clearance through the liver, with subsequent clearance via the kidneys (Figure S6A). Magnetic resonance imaging (MRI) can visualize the distribution of iron in tissues, allowing us to track the accumulation of IONP6 in the ischemic brain over time. The images showed that the IONP6 concentration in the brain increased over time (Figure 3A). At 12 h post‐injection, the brain accumulation of IONP6 reached 8% ID/g, which was about 2 times that of the 0 h time point (Figure 3B). In addition, we also observed that the IONP6 accumulation reached a peak at 24 h after injection. The IONP6 content in the ischemic hemisphere of the pMCAO rats was approximately 24 mg/kg (Figure S6B). However, no significant difference was observed between the 0 and 72 h time points, suggesting that IONP6 has been largely metabolized from the brain by 72 h (Figure 3B). These experiments supported that the IONP6 was accumulated in the brain with a relatively higher dosage compared with other previous studies [39]. Ischemia and inflammation lead to the leakage of the BBB [40], which may be the reason for the accumulation of IONP6 in the brain.

### 2.6 Regulation of Neuroinflammatory Response by IONP6 in Ischemic Stroke

We next examined the effect of IONP6 on the activation state of the recruited host immune cells in ischemic stroke rats, which was monitored by the microglial pro‐inflammatory marker (iNOS) and anti‐inflammatory marker (ARG1). IONP6 group (5 mg/kg) distinctly reduced the expression of iNOS in stroke rats. In contrast, the protein expression for ARG1 remained significantly different. The expression level of ARG1 in the 5 mg/kg IONP6 group was 3 times higher than that in the pMCAO group. However, there was no significant difference in the expression of iNOS and ARG1 protein between the model group and the 2.5 mg/kg IONP6 group (Figure 3C,D). Similarly, IONP6 treatment induced a significant decrease in the iNOS fluorescence signal and an increase in the ARG1 fluorescence signal in stroke rats (Figure 3E,F). Collectively, these findings suggest that ischemic infarction induces microglial activation and promotes their over‐activation, while the 5 mg/kg IONP6 treatment group appeared to mitigate inflammation in ischemic stroke.

To further assess whether the IONP6 possessed the anti‐inflammatory effects we described above, the in vivo level of various inflammatory cytokines was measured in the penumbra brain region after ischemic stroke for 24 h. As shown in Figure 3G, IONP6 significantly downregulated the mRNA expression levels of the pro‐inflammatory cytokines *Il‐1β*, *Il‐6* and *Tnf‐α*, while the anti‐inflammatory marker *Arg1* was elevated significantly [41]. These data indicate that IONP6 induces the conversion of the microglia from an M1‐like phenotype to an M2‐like phenotype and effectively suppresses the secretion of pro‐inflammatory factors in pMCAO rats.

**FIGURE 4:**
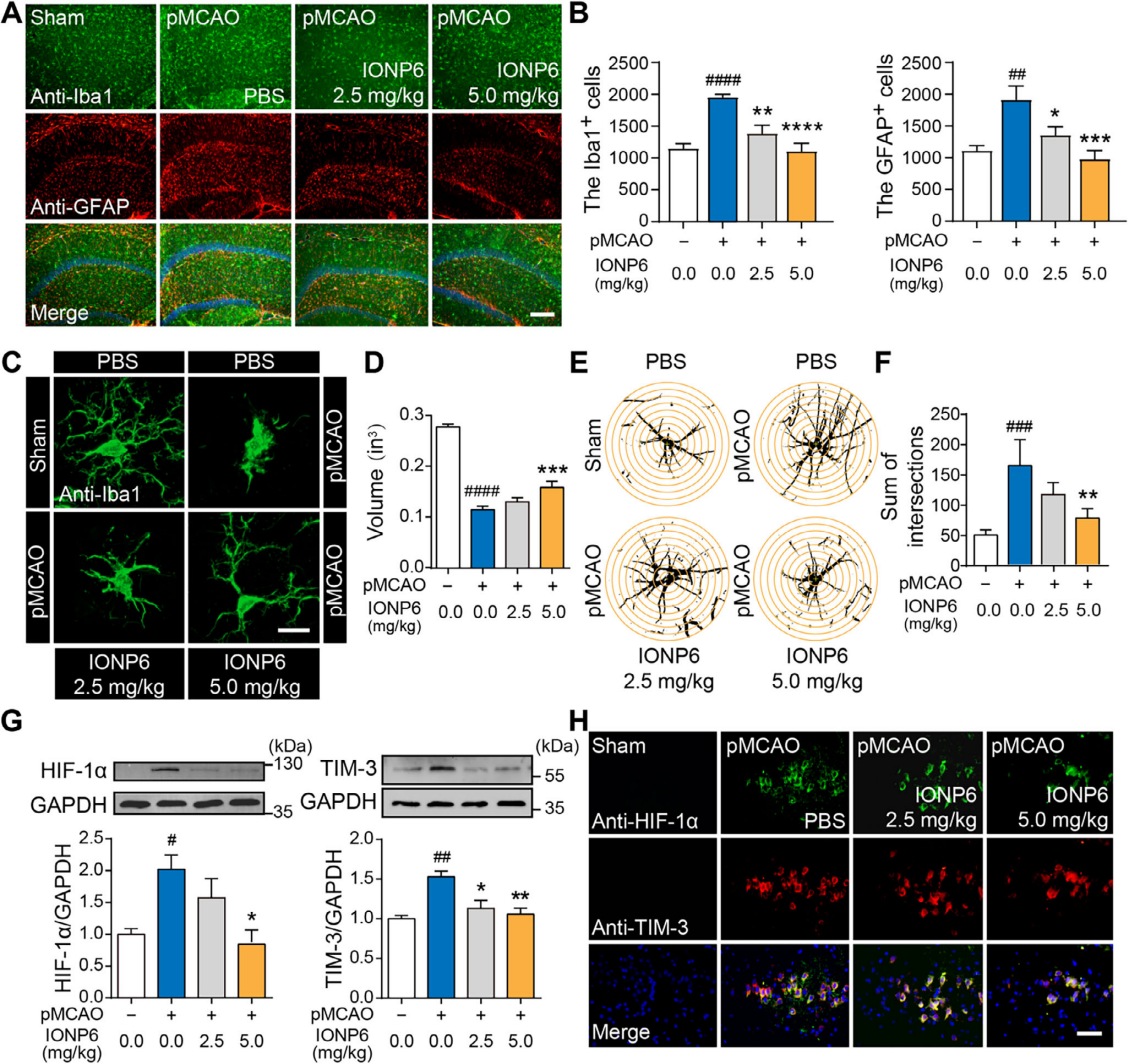
IONP6 inhibited microglial and astrocyte over‐activation and attenuated inflammatory responses via the HIF‐1α/TIM‐3 axis in pMCAO rats. (A) Immunofluorescent staining of microglia (anti‐Iba1 labeled, green), astrocytes (anti‐GFAP labeled, red) in the hippocampus of brains from sham rats, pMCAO rats with or without IONP6 treatment. Scale bar, 50 µm. (B) Quantitation of Iba1^+^ cells (left) and GFAP^+^ cells (right). (A,B) demonstrated that IONP6 reduced the activation of microglia and astrocytes (n = 7 independent slices from 3 rats for pMCAO and 2.5 mg/kg IONP6 group, n = 8 independent slices from 3 rats for Sham and 5 mg/kg IONP6 group). (C) The morphology of microglia (anti‐Iba1 labeled, green) in the hippocampus of brains from sham rats, pMCAO rats with or without IONP6 treatment. Scale bar, 20 µm. (D) Quantitative analysis of microglial volume from different groups in panel C (n = 4 independent slices from 3 rats). (E) Representative images for Sholl analysis showing astrocyte morphology in the hippocampus, derived from the images in Figure S8A. The interval of the concentric circles was 0.1 in. (F) Analysis of the number of process intersections in GFAP^+^ astrocyte in the hippocampus of brains from sham rats, pMCAO rats with or without IONP6 treatment (n = 3 independent slices from 3 rats). (C–F) demonstrated that IONP6 maintained the normal morphology of microglia and astrocytes in pMCAO rats. (G) Western blot analysis of HIF‐1α (left) and TIM‐3 (right) protein in brains from sham rats, pMCAO rats with or without IONP6 treatment (n = 3 rats per group). (H) Immunohistochemistry was performed in the hippocampus of brains from sham rats and pMCAO rats, with or without IONP6 treatment, using anti‐HIF‐1α (green) and anti‐TIM‐3 (red) antibodies. Scale bar, 12.5 µm. (G,H) demonstrated that IONP6 attenuated inflammatory responses via HIF‐1α/TIM‐3 axis in pMCAO rats. The data are presented as the mean ± SEM. *p*‐values are calculated by one‐way ANOVA. Rats without IONP6 treatment served as the sham group, pMCAO rats without IONP6 treatment served as the pMCAO group, and pMCAO rats with IONP6 treatment served as the IONP6 group. ^##^ *p*< 0.01, ^###^ *p*< 0.001, ^####^ *p*< 0.0001, pMCAO group vs. the sham group, ^*^ *p*< 0.05, ^**^ *p*< 0.01, ^***^ *p*< 0.001, ^****^ *p*< 0.0001, 2.5 or 5 mg/kg IONP6 group vs. the pMCAO group.

After investigating the effects of IONPs on cell states in pMCAO rats, we further analyzed two major cell populations, microglia and astrocytes, that drive neuroinflammation and are highly abundant in the cortex and hippocampus regions of the brain [42]. The increased expression of Iba1 (ionized calcium‐binding adaptor molecule 1, a microglial marker) [43] and GFAP (glial fibrillary acidic protein, an astrocyte marker) [42, 44], induced by pMCAO, was attenuated by IONP6 treatment in both the hippocampus (Figure 4A,B) and cortex (Figure S7). It is well documented that IONP6 can reduce the activation of microglia and astrocytes in pMCAO rats. Under normal conditions, resting microglia are small, highly branched, and evenly distributed throughout the brain parenchyma. When the brain homeostasis is broken, such as central nervous system injury or infection, microglia are induced, and their branches begin to retract [45]. Figure 4C showed the morphology of Iba1‐labeled microglia, which were small and had sufficient branches in the sham‐operated group, while the enlarged bodies and the branches of microglia almost disappeared in the pMCAO group. The branches of microglia in the IONP6 group increased, especially in the 5 mg/kg group, and the morphology of microglia changed significantly, returning to the resting state. Then we counted the volume of microglia, observing that the volume of microglia in the model group became smaller, and the volume of microglia in the rat brain treated with IONP6 became larger (Figure 4D). We also observed astrocytes with the GFAP antibody and performed a Sholl analysis to define the changes in their morphology (Figure 4E; Figure S8A). We measured the level of process ramification, the sum of intersects, and the ending radius, which were all significantly increased in the pMCAO rats (Figure 4F; Figure S8B). Excitedly, treatment with 5 mg/kg IONP6 could improve the above‐mentioned astrocyte indexes. These aspects suggest that IONP6 contributes to reversing the over‐activation of microglia and astrocytes in ischemic stroke.

### 2.7 Inhibition of Inflammation Through the HIF‐1α/TIM‐3 Axis by IONPs

Emerging evidence has revealed that hypoxia inducible factor‐1 alpha (HIF‐1α), a transcription factor, can be upregulated and stabilized through NO‐mediated S‐nitrosylation [46]. Interestingly, recent findings reveal that hypoxia‐ischemia (H/I) injury in adult mice increases the expression of the T cell immunoglobulin domain and mucin domain‐3 (TIM‐3) in activated microglia, which is mediated by the HIF‐1α [47]. Moreover, it has been proven that negative regulation of TIM‐3 can polarize microglia into an anti‐inflammatory phenotype and inhibit the secretion of inflammatory factors [48]. To determine the mechanisms underlying the above effects of IONP6, we continued to verify the effect of IONP6 on regulating the HIF‐1α/TIM‐3 axis. Western blot analysis demonstrated that the expression levels of TIM‐3 were increased in OGD‐induced BV‐2 cells (Figure S9A). Importantly, the expression levels of HIF‐1α and TIM‐3 in the IONP6‐treated group were significantly lower than those in the OGD group. The mRNA levels of *Hif‐1α* and *Tim‐3* showed similar trends to their protein levels (Figure S9B,C). Furthermore, the expression of HIF‐1α and TIM‐3 in the brain parenchyma of the pMCAO rats with or without IONP6 treatment was consistent with the in vitro experiment (Figure 4G), which was also confirmed by immunofluorescence staining (Figure 4H; Figure S10). Taken together, these results suggest that IONP6 downregulates TIM‐3 expression in a HIF‐1‐dependent manner under OGD, exerting anti‐inflammatory effects by suppressing the HIF‐1α/TIM‐3 axis. This further inhibits the secretion of pro‐inflammatory mediators and cytokines induced by ischemic injury.

### 2.8 Behavioral and Neurochemical Evaluations of IONP6 Treatment in pMCAO Rats

**FIGURE 5:**
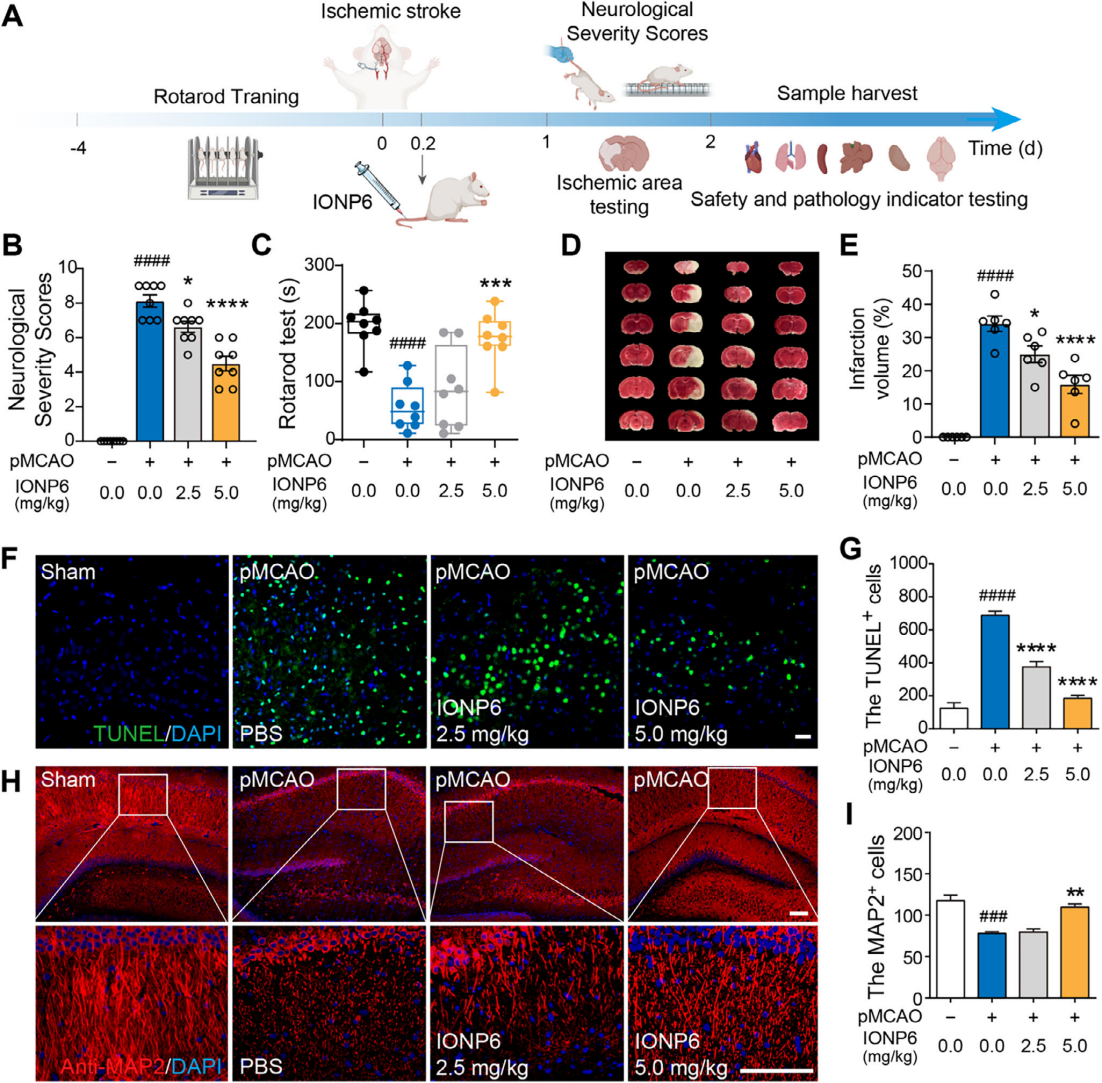
IONP6 protected against brain injury induced by ischemic stroke. (A) Schematic of the experimental process. pMCAO rats were treated with 2.5 or 5 mg/kg IONP6 by intravenous injection. Rats were subjected to neurological severity score and rotarod tests after 24 h. (B,C) Quantitative analysis of the Neurological test (B) and forced rotarod test (C). Neurological tests demonstrated that IONP6‐treated rats exhibited a significant reduction in neurological damage (B, n = 8 rats per group). Moreover, IONP6‐treated rats showed a marked improvement in motor coordination and integration on the rotarod test compared to pMCAO rats (C, n = 8 rats per group). (D,E) Representative images of TTC‐stained coronal brain slides (D) and quantitative analysis of infarct volume in each group (E) (n = 6 rats per group). Coronal brain slides were arranged in sequence. White areas in the ipsilateral hemisphere represented the infarcted regions; IONP6 reduced the infarct volume in stroke rats. (F) Representative images of TUNEL‐stained cells in the hippocampus of brains. Scale bar, 12.5 µm. (G) Quantitative analysis of TUNEL^+^ cells in panel F (n = 4 independent slices from 3 rats). (H) The dendrite of a neuron was observed by MAP2 staining in the hippocampus of the brain. Scale bar, 12.5 µm. (I) Quantitative analysis of MAP2^+^ cells in panel H (n = 4 independent slices from 3 rats). The data are presented as the mean ± SEM. *p*‐values are calculated by one‐way ANOVA. ^###^ *p*< 0.001, ^####^ *p*< 0.0001, pMCAO group vs. the sham group, ^*^ *p*< 0.05, ^**^ *p*< 0.01, ^***^ *p*< 0.001, ^****^ *p*< 0.0001, 2.5 or 5 mg/kg IONP6 group vs. the pMCAO group.

Inspired by the anti‐inflammatory effects of IONP6 in vivo, we next investigated potential improvements of IONP6 in altering behavioral and neuroanatomical deterioration relevant to ischemic stroke. IONP6 was singly injected intravenously after the completion of the pMCAO model. After being injected for 24 h, the behavioral evaluation of rats was carried out, including neurological severity scores and the rotarod test (Figure 5A). The neurological severity scores and the average scores were calculated (Figure 5B), suggesting that treatment with IONP6 significantly ameliorated ischemia‐induced neurological deficits. The rotarod test is performed to test the deficits of motor balance and locomotor coordination in pMCAO rats [49]. Administration of IONP6 for 5 mg/kg remarkably prolonged the latency time to fall from the rod (Figure 5C). TTC staining was used to visualize brain infarcts, and infarct size was quantified from coronal sections in pMCAO rats treated with or without IONP6. IONP6 treatment of 5 mg/kg decreased the brain infarct size, but only a slight reduction in infarct size in the 2.5 mg/kg IONP6 treatment group (Figure 5D,E).

Dendrites are essential components of neurons, playing a key role in receiving signals from other neurons and facilitating communication within the neural network [50, 51]. Under normal conditions, dendrites maintain their structural integrity, which is vital for proper neuronal function. However, in ischemic stroke, neuronal damage often leads to dendritic degeneration and loss, contributing to impaired neural activity and cognitive function [52]. Initially, we observed neuronal apoptosis using H&E staining and TUNEL staining to assess the effects of IONP6 treatment on neuronal damage. H&E staining results showed a significant increase in neuronal apoptosis in both the cerebral cortex and hippocampal regions in the model group compared to the sham group, as illustrated in Figure S11. The protective effects of IONP6 were further assessed by examining apoptosis in the hippocampus, the region critical for neurogenesis. TUNEL staining revealed a marked decrease in TUNEL^+^ cells in the IONP6 group, reversing the apoptosis trend (Figure 5F,G). These findings suggest that 5 mg/kg IONP6 effectively prevents ischemia‐induced neuronal damage. To further explore whether the attenuation of microglia‐related neuroinflammation by IONP6 enhances neuronal recovery, we examine dendritic changes in the hippocampus, which plays a central role in synaptic plasticity. As described in previous studies [53], dendritic degeneration in the pMCAO group was marked by the truncation of dendritic processes and a significant loss of dendritic branching, indicative of neuronal damage and impaired synaptic connectivity following ischemic injury. In contrast, rats treated with IONP6 exhibited notable preservation and restoration of dendritic structure, with a reduction in dendritic truncation and an increase in the number of neurons, suggesting a recovery of synaptic integrity and neuronal function. The 2.5 mg/kg IONP6‐treated group also demonstrated moderate improvements in dendritic structure, though the effects were less substantial than those observed at the higher dose 5 mg/kg (Figure 5H,I; Figure S12). These findings highlight the dose‐dependent efficacy of IONP6 in promoting neuronal recovery, with higher doses yielding more significant restoration of dendritic integrity, supporting its potential as a therapeutic agent for synaptic repair following ischemic stroke.

Although IONPs have a wide range of biological applications and good biocompatibility, the security in ischemic stroke is not fully understood. Here, we observed the histopathological state of the organs of SD rats treated with 2.5 and 5 mg/kg IONP6 after ischemic stroke. After 24 h of IONP6 administration, H&E staining showed no abnormal changes in major organs (Figure S13). This indicates that the short‐term efficacy and safety of IONP6 can be ensured, but its long‐term safety and efficacy remain an important area for further investigation and warrant additional research in future studies. In this study, IONP6 exerts its anti‐inflammatory effects by scavenging RONS, particularly NO, which leads to the downregulation of HIF‐1α. Reduced HIF‐1α levels subsequently downregulate TIM‐3, a negative regulator of immune responses. The downregulation of TIM‐3 promotes the polarization of microglia from the pro‐inflammatory M1 phenotype to the neuroprotective M2 phenotype, thereby resolving inflammation and supporting tissue repair.

## 3 Conclusions

While other iron oxide‐based nanozymes have shown potential in scavenging ROS and promoting neuroprotection in stroke models, IONP6 offers distinct advantages over these traditional nanozymes. Unlike most nanozymes, which are primarily designed to scavenge ROS, IONP6 additionally targets NO, a critical contributor to stroke‐induced damage. This makes IONP6 a comprehensive solution that can address both oxidative stress and NO‐induced neuroinflammation, which are two central mechanisms in stroke pathology. The unique ability of IONP6 to bind with NO and form stable complexes sets it apart from traditional nanozymes, which cannot target NO. This mechanism not only eliminates NO but also prevents the formation of its more damaging derivative, ONOO^−^, which is a known culprit in BBB disruption and neuronal death. DHCA‐modified IONP6 can directly convert harmful RONS (•O_2_^−^, H_2_O_2_) into O_2_ urgently required by ischemic brain cells, thereby exerting neuroprotective effects and potentially extending the therapeutic window for stroke treatment. Another key advantage of IONP6 is its ability to regulate microglial polarization, a feature not commonly associated with traditional nanozymes. By promoting the shift from the pro‐inflammatory M1 phenotype to the anti‐inflammatory M2 phenotype, IONP6 directly addresses the immune response, fostering tissue repair and neuroprotection in ischemic stroke. Thus, IONP6 combines the traditional advantages of nanozymes (such as ROS scavenging) with novel mechanisms for RNS elimination and microglia modulation, providing a multifaceted approach to treating ischemic stroke that is superior to current therapies based on either ROS scavenging or inflammation modulation alone.

While this study demonstrates the therapeutic potential of IONP6 in modulating neuroinflammation and microglial polarization in ischemic stroke, several avenues for future research remain. First, it is essential to investigate the long‐term therapeutic effects of IONP6, including its chronic exposure and sustained administration in the ischemic stroke model, to evaluate its long‐term safety and efficacy. Further studies will also be necessary to assess the translatability of IONP6 in larger animal models, including non‐human primates, to evaluate its potential for clinical application. Lastly, examining the synergistic effects of IONP6 in combination with other established therapies, such as thrombolytic agents or neuroprotective drugs, may provide insights into its potential as part of a multimodal therapeutic approach to improve stroke recovery.

## 4 Experimental Section

### 4.1 Materials

Ferric chloride hexahydrate (>99%), sodium oleate were purchased from Sinopharm Chemical Reagent Co., Ltd. Oleic acid, benzyl ether, 1‐octadecene and were purchased from Sigma–Aldrich Co., Ltd. Oleyl alcohol (65.0%) were purchased from TCI Co., Ltd.

### 4.2 Syntheses of the IONPs

First, an iron‐oleate complex was synthesized according to the synthesis method mentioned above. Briefly, A mixture of 20 mL ethanol, 15 mL distilled water, and 35 mL hexane was prepared, and then 2.7 g ferric chloride (FeCl_3_·6H_2_O, 10 mmol) and 9.1 g sodium oleate (30 mmol) were added. The solution was heated to 70°C for 4 h. The iron–oleate complex exists in the upper organic phase and was washed with distilled water. Finally, the iron–oleate complex in the form of a wax solid was obtained.

#### 4.2.1 Synthesis of IONP3

IONP3 were synthesized according to a previous report [30]. At room temperature, 2 mmol iron oleate complex (1.8 g), 12 mmol oleanol (3.2 g), and 10 g benzyl ether were mixed. The mixture was then heated to 200°C at a constant heating rate of 10°C min^−1^ under an inert atmosphere. When the temperature rose to 200°C, the reaction solution was rapidly cooled to room temperature. The nanoparticles were precipitated with acetone, centrifuged, and dispersed with hexane.

IONP6, IONP10, and IONP16 were synthesized by the heat‐up process. To synthesize the nanocrystals of 6 nm in diameter, the 2 mmol iron‐oleate complex (1.8 g) and 0.3 g of oleic acid (1 mM) were dissolved in 10 g of 1‐octadecene at room temperature. The reaction mixture was heated to 280°C with a constant heating rate of 3.3°C min^−1^, and then kept at that temperature for 30 min. At this time, the solution changed from clear to dark brown. The resulting solution containing the nanocrystals was then cooled to room temperature, and ethanol was added to the solution to precipitate the nanocrystals. Larger nanocrystals (10, 16 nm) were synthesized by increasing the ratio between oleic acid and iron precursor concentration, and increasing the temperature from 300°C to 320°C.

The surface modification was performed using a ligand‐exchange reaction [54]. A total of 50 mg of 3, 4‐dihydroxyhydrocinnamic acid (DHCA) was dissolved in 6 mL of THF in a three‐neck flask (25 mL). The resulting solution was heated to 50°C under argon flow. Then, 20 mg of hydrophobic magnetic nanoparticles dispersed in 1 mL of THF were added dropwise. After 3 h, the reaction was cooled to room temperature, and 500 µL NaOH (0.5 ‐m) was introduced to the solution to precipitate the magnetic nanoparticles. The precipitate was collected by centrifugation (3000 rpm/min) and redispersed in water for further use.

### 4.3 Characterization

Transmission electron microscopy on a Talos F200x electron microscope (FEI) was used to detect morphology with a voltage of 200 kV. The XRD was recorded by a Bruker D8 Advanced Diffractometer System with a Cu Kα radiation target. X‐ray photoelectron spectroscopy (XPS, PHI5000 VersaProbe III, ULVAC‐PHI Inc.) was conducted to measure the chemical states of the samples. FT‐IR spectra were collected using a Tensor27 (Bruker) spectrometer. Hydrodynamic size was tested by dynamic light scattering (DLS, Malvern Zetasizer nano‐ZS instrument). Inductively coupled plasma optical emission spectrometry (Thermo Fisher, Solaar M5 Series) was used to analyze Fe concentrations of samples. The confocal laser scanning microscopy (CLSM) images were recorded on TCS SP8 (Leica, Germany) and LSM 800 (Zeiss, Germany).

### 4.4 Verification of NO and RNS Clearance by IONPs

We used the DPPH• (2,2‐di‐(4‐tert‐octylphenyl)‐1‐picrylhydrazyl radical) assay to detect the scavenging activity of IONPs on RNS. DPPH• (40 µg/mL) and IONPs (100 µg/mL) were mixed in the same proportion, and the reaction time was 0.5 h in the dark at room temperature. Then the absorbance was recorded at 517 nm.

An electron paramagnetic resonance (EPR) spectrometer was used to detect the NO scavenging activity of IONPs. The donor of NO is NOC7. Carboxy‐PTIO with five‐line EPR signals reacts with NO to generate Carboxy‐PTI with seven‐line EPR signals. The testing solution contained 10 µM carboxy‐PTIO and 250 µM NOC7. EPR spectra were recorded in the absence or presence of 100 µg/mL IONPs.

For XPS sample processing, 2 mL IONPs (1 mg/mL) were added to the two‐neck flask. The flask was then charged with NO (60 mL/min) at 20°C for 1 h. Finally, the products were collected and dried.

The catalytic activity of IONP6 against H_2_O_2_ was characterized by monitoring the absorbance change at 240 nm. In a typical assay (0.1 mL), 20 µL IONP6 (0.0, 0.5, 1.0, 1.5, 2.0, 4.0, 6.0 mg/mL) or the same amount of PBS was added into a solution of H_2_O_2_ (6 mM) in PBS buffer. The absorbance at 240 nm was monitored immediately and measured at 0 min and 150 min. 20 µL PBS without any nanocatalysts was used as a control. The residual H_2_O_2_ was quantified by measuring the absorbance difference between 0 and 150 min.

The SOD activity of IONP6 was quantified with the superoxide dismutase activity kit (boxbio, AKAO001M), according to the manufacturer's protocol. The inhibition rate and SOD activity were calculated from the absorbance at 560 nm.

### 4.5 Cell Culture

BV‐2 cells, previously characterized as an immortalized murine microglial cell line, were cultured in DMEM containing 10% FBS, 100 units/mL penicillin, and 100 µg/mL streptomycin in a humidified atmosphere with 5% CO_2_.

### 4.6 Oxygen‐Glucose Deprivation

The oxygen‐glucose deprivation (OGD) model was established as described previously with minor modifications. Briefly, 12 h after plating, the BV‐2 cells were washed with glucose‐free DMEM (Gibco, USA) and incubated with glucose‐free DMEM or glucose‐free DMEM containing 25 µg/mL IONP6. They were further incubated in an anaerobic chamber containing a mixture of 5% CO_2_ and 95% N_2_ at 37°C for 6 h. Then, the cultures were switched back to their original culture condition for 1 h.

### 4.7 Cell Viability

The MTT assay was used to assess the viability of BV‐2 after treatment with IONPs in a 96‐well plate. After 24 h of stimulation, MTT reagent (M8180, Solarbio, China) was added directly to the wells of the 96‐well plate, which was planted with BV‐2 cells. The cells were incubated for a further 4 h. Then, DMSO was added to the pore plate to dissolve the purple formazan, and the absorbance value was measured at 570 nm using a plate reader (SpectraMax i3x, MD), and viability was calculated as % viability compared to control medium.

### 4.8 NO Content Detection

Nitrite is the oxidation product of NO. The diazotization reaction between NO_2_^−^ and p‐aminobenzenesulfonic acid could be detected by the Griess reagent. The reaction was coupled with N‐(1‐naphthyl)‐ethylenediamine to form a yellow product. The absorbance value of the product at 540 nm (in direct proportion to the content of NO_2_^−^) was determined, and the amount of NO produced was determined accordingly. The absorbance value of the BV‐2 cell medium was detected after treatment with IONPs in combination with LPS (L4516, Sigma) or OGD. After 24 h of stimulation, Griess reagent was mixed with the medium in a ratio of 1:1, and then the absorbance value at 540 nm was measured. The formula of NO_2_^−^ content is NO_2_^−^ (µmol/L) = (OD – 0.33)/0.0099.

### 4.9 Flow Cytometry

For staining of GFAP, cells were fixed for 20 min with PFA and permeabilized for 5 min with digitoxin (50 µg/mL), washed twice with PBS, incubated with anti‐iNOS (CST, 13120, 1:100), anti‐ARG1 (Abcam, ab239731, 1:100) in PBS for 30 min, and stained with TRITC and FITC fluorescent dye‐conjugated secondary antibody (1:200, Jackson Laboratories) for 30 min. The data were analyzed with the Cell Quest software (BD Bioscience) and FlowJo software packages.

### 4.10 Animal

Adult male Sprague–Dawley rats, purchased from Beijing Vital River Laboratory Animal Technology Co., Ltd., 220–270 g at the time of surgery and 4–5 per cage under standard feeding conditions, free to eat and drink. All procedures and treatments were conducted according to the Regulations for the Administration of Affairs Concerning Experimental Animals (Tianjin, revised in June 2004) and the Animal Ethics Committee of Nankai University (approval number: 2021‐SYDWLL‐000457).

### 4.11 Rat Permanent Cerebral Ischemia Model and Drug Administration

Rats were anesthetized with 3.5% chloralhydrate, fixed on the back, shaved, and the midline of the neck was cut open. The right common carotid artery (CCA), external carotid artery (ECA), and internal carotid artery (ICA) were isolated. The distal CCA and the proximal ECA were ligated. At the distal end of the CCA, a paraffin‐embedded 5 cm line (0.26 mm in diameter) was inserted into the right ICA, about 16 mm to block the middle cerebral artery. The sham group received the same surgical procedure, but the fish line was not inserted into the middle cerebral artery. IONP6 or PBS was administered to the rats approximately 3 h after the completion of the pMCAO surgery, once the rats had recovered from anesthesia. The sham operation group and the model group were intravenously injected with PBS, and the administration group was injected with IONP6 of 2.5 and 5 mg/kg.

### 4.12 Toxicity Assay In Vivo

Healthy Sprague–Dawley rats were divided into four groups: sham, pMCAO, 2.5, and 5 mg/kg. 24 h after injection of IONP6, the organs (brain, heart, kidney, liver, spleen, lung) for histological analysis via H&E staining were harvested from the rats.

### 4.13 Distribution of IONP6 In Vivo

After ischemic stroke in SD rats, IONP6 was injected into the two groups of rats by the tail vein with different doses of 0 and 5 mg/kg. After 0, 12, 24, and 72 h, the brains, hearts, kidneys, livers, spleens, and lungs were rapidly acquired and weighed. The tissue samples were digested with nitric acid to obtain a clear solution, and then the iron content was measured by ICP‐OES.

### 4.14 TTC Staining

After 24 h of administration of IONP6, the rats were killed by cervical dislocation. And the brain tissue was taken out and frozen at −20°C. The brain was cut into 6 consecutive 2 mm‐thick coronal sections, immersed in 2, 3, 5‐triphenyltetrazolium chloride (Sigma, T8877‐10G) solution for 30 min at 37°C, fixed in 4% paraformaldehyde, photographed, and the infarct volume was analyzed using Image Pro Plus software.

### 4.15 Behavioral Testing

#### 4.15.1 Rotarod Test

The performance of the rotating rod was evaluated on a suspension rod of an accelerated rotating rod device (diameter: 9 cm), and the rotating rod device was accelerated at a constant rate of 0 to 50 rpm for 300 s. Rats were continuously trained for 5 days and placed on a stick for 3 trials. Record the time of each trial. The test ended when the mouse was detached from the rotating rod or the time reached 300 s. A break of 300 s was allowed between each test.

#### 4.15.2 Neurological Severity Scores

To assess the recovery of neurological function in rats after administration, neurological function was assessed using NSS. NSS is a combination of exercise, sensory, balance, and reflex tests with a score range of 0–18 (normal score 0, maximum defect score 18). Higher scores indicate more severe neurobehavioral disorders.

### 4.16 Immunohistochemistry

The rats were anesthetized, perfused with 0.9% saline, and fixed with 4% paraformaldehyde. Brain tissue was collected, fixed in 4% paraformaldehyde for 24 h, dehydrated, waxed, and transparent paraffin sections, with a thickness of 20 µm. Sections were incubated with goat serum containing 0.4% Triton X‐100 for 1 h, and then sections were incubated overnight with anti‐Iba1 (WaKo, Cat 019–19741, 1:200) antibody, anti‐GFAP (CST, 3670, 1:300) antibody, anti‐MAP2 (Abcam, ab32454, 1:200) antibody, anti‐ARG1 (Abcam, ab239731, 1:200) antibody, anti‐iNOS (CAYMAN, 160862, 1:200) antibody, anti‐HIF‐1α (Abcam, ab8366, 1:200) antibody and anti‐TIM‐3 (ABclonal, A2516, 1:200) antibody at 4°C. After washing with TBST, sections were incubated with FITC and TRITC fluorescent dye‐conjugated secondary antibody (1:200, Jackson Laboratories) for 1 h. Finally, the nuclei were stained with DAPI staining solution (VECTOR LABORATORIES, H‐1200). Images were obtained by inverted fluorescence microscopy, and ImageJ software was used to analyze the images.

### 4.17 Analysis of Microglia and Astrocyte Morphology

Microglia morphology was assessed using FIJI software. Basic shape descriptors, such as the area, were performed with the plugin Shape Descriptors. Confocal images immunostained with GFAP antibody were serially stacked and projected maximally. The maximal projection image of the GFAP signal in the hippocampus was adopted for analysis. The plugin of Sholl analysis applied in FIJI automatically draws serial concentric circles at 0.1‐in intervals from the center of the DAPI signal to the end of the most distant process in every single astrocyte and analyzes the number of intercepts of GFAP processes in each circle and the ramification index.

### 4.18 Western Blot

Centrifuged the cell or rat brain lysate and took the supernatant. The protein concentration was measured by the Bradford method. Add 4×SDS at 95°C for 10 min. An equal amount of protein (20 µg) was loaded onto an 8%, 10% gel according to the molecular weight of the target protein. The protein was then separated using SDS‐PAGE, transferred to a polyvinylidene fluoride membrane (PVDF), and blocked with 5% BSA for 90 min. The following primary antibodies were then incubated overnight at 4°C, anti‐iNOS (CST, 13120, 1:1000), anti‐ARG1 (Abcam, ab239731, 1:1000), anti‐HIF‐1α (Abcam, ab179483, 1:1000), anti‐TIM‐3 (ABclonal, A2516, 1:1000), anti‐GAPDH (EASYBIO, BE0023, 1:2000) and mouse anti‐β‐Actin (EASYBIO, BE0021, 1:2000). The membrane was then incubated with anti‐mouse secondary antibody (EASYBIO, BE‐2004‐100, 1:5000), anti‐rabbit secondary antibody (1:5000, BE2005‐100) for 1 h at room temperature. Immunoreactivity was detected using a Millipore ECL substrate (Millipore, WBKLS0500).

### 4.19 RNA Extraction and RT‐qPCR

Total RNA from cells or tissues was extracted using TRIzol reagent (Invitrogen, 15596026), and the concentration and purity of the RNA were determined using NanoDrop (Thermo Fisher Scientific). Reverse transcription of RNA to complementary DNA was performed using a TransScript First‐Strand cDNA Synthesis kit (AT301‐02, Transgen). The mRNA levels of *Tnf‐α*, *Arg1*, *Il‐6*, *Il‐10*, *Il‐4*, *Il‐1β*, *Hif‐1α*, and *Tim‐3* were quantified by SYBR Green‐based RT‐qPCR kit (TIANGEN, FP205‐02), and data were quantified using the ΔΔCt method, and *β‐actin* expression was performed for standardization.

### 4.20 Statistical Analysis

Statistical analyses were conducted via GraphPad Prism 8.3 software. Statistical comparisons between the two groups were assessed using an unpaired *t*‐test. For multiple comparisons, statistical significance was determined by one‐way analysis of variance (ANOVA). Statistical differences in organ distribution data were analyzed by two‐way ANOVA and unpaired t‐test. Statistical significance was indicated as ^*^ *p* < 0.05, ^**^ *p* < 0.01, ^***^ *p* < 0.001, and ^****^ *p* < 0.0001, ^#^ *p* < 0.05, ^##^ *p* < 0.01, ^###^ *p* < 0.001, and ^####^ *p* < 0.0001. All data were presented as mean ± standard error of the mean (SEM).

## Author Contributions

X.C., H.F., and X.X. conceived and designed the study, performed the analysis, and wrote the paper. Y.M., C.W., and Y.Q. designed and performed experiments and performed analysis. M.L. and G.L. synthesized and characterized the materials. J.L., Z.Y., and F.X. designed and performed animal experiments. W.H., G.L., D.S., and M.L. analyzed the data and participated in the discussion. All authors have approved the final version of the manuscript.

## Conflicts of Interest

The authors declare no competing financial interests.

## Supporting information




**Supporting file**: advs73631‐sup‐0001‐SuppMat.docx.

## Data Availability

The data that support the findings of this study are available from the corresponding author upon reasonable request.
