# Supplementary material for: Iron Oxide Nanozyme as Reactive Oxygen and Nitrogen Species Scavenger to Regulate Microglial Homeostasis in Stroke
Source: Adv Sci (Weinh). 2026 Jan 20;13(18):e18191. doi: 10.1002/advs.202518191 (PMC13042548; doi:10.1002/advs.202518191)
Supplement: Supplementary file 1 — Supporting file: advs73631‐sup‐0001‐SuppMat.docx. [file ADVS-13-e18191-s001.docx]

# Supporting Information

**Iron Oxide Nanozyme as Reactive Oxygen and Nitrogen Species Scavenger to Regulate Microglial Homeostasis in Stroke**

Yilin Qi^1, ‡^, Chunxiao Wang^1, ‡^, Yuqing Miao^2,3 ‡^, Jiamin Li^1^, Zengyu Xun^1^, Di Sun^1^, Fei Xu^1^, Minrui Liu^2^, Heping Wang^4^, Galong Li^2,5^, Xuyi Chen^6,*^, Haiming Fan^2, *^ ,and Xue Xue^1,7, *^

^1^State Key Laboratory of Medicinal Chemical Biology, College of Pharmacy, Nankai University, Haihe Education Park, 38 Tongyan Road, Tianjin, 300353, China

^2^Key Laboratory of Synthetic and Natural Functional Molecule of the Ministry of Education, College of Chemistry and Materials Science, Northwest University, Xi'an, 710069, China

^3^Institute for Chinese Medicine Frontier Interdisciplinary Science and Technology, Shaanxi University of Chinese Medicine, Shaanxi, 712046, China

^4^State Key Laboratory of Advanced Medical Materials and Devices, Tianjin Institutes of Health Science, Institute of Radiation Medicine, Chinese Academy of Medical Sciences & Peking Union Medical College, Tianjin, 300192, China

^5^School of Biomedical Engineering, Air Force Medical University, Xi'an, 710032, China

^6^Characteristic Medical Center of People’s Armed Police Forces, Tianjin, 300353, China

^7^Academy for Advanced Interdisciplinary Studies, Nankai University, Tianjin, 300353, China

^‡^These authors contributed equally to this work.

*Corresponding author.

Email: [chenxuyi1979@126.com,](mailto:chenxuyi1979@126.com,) fanhm@nwu.edu.cn, xuexue@nankai.edu.cn


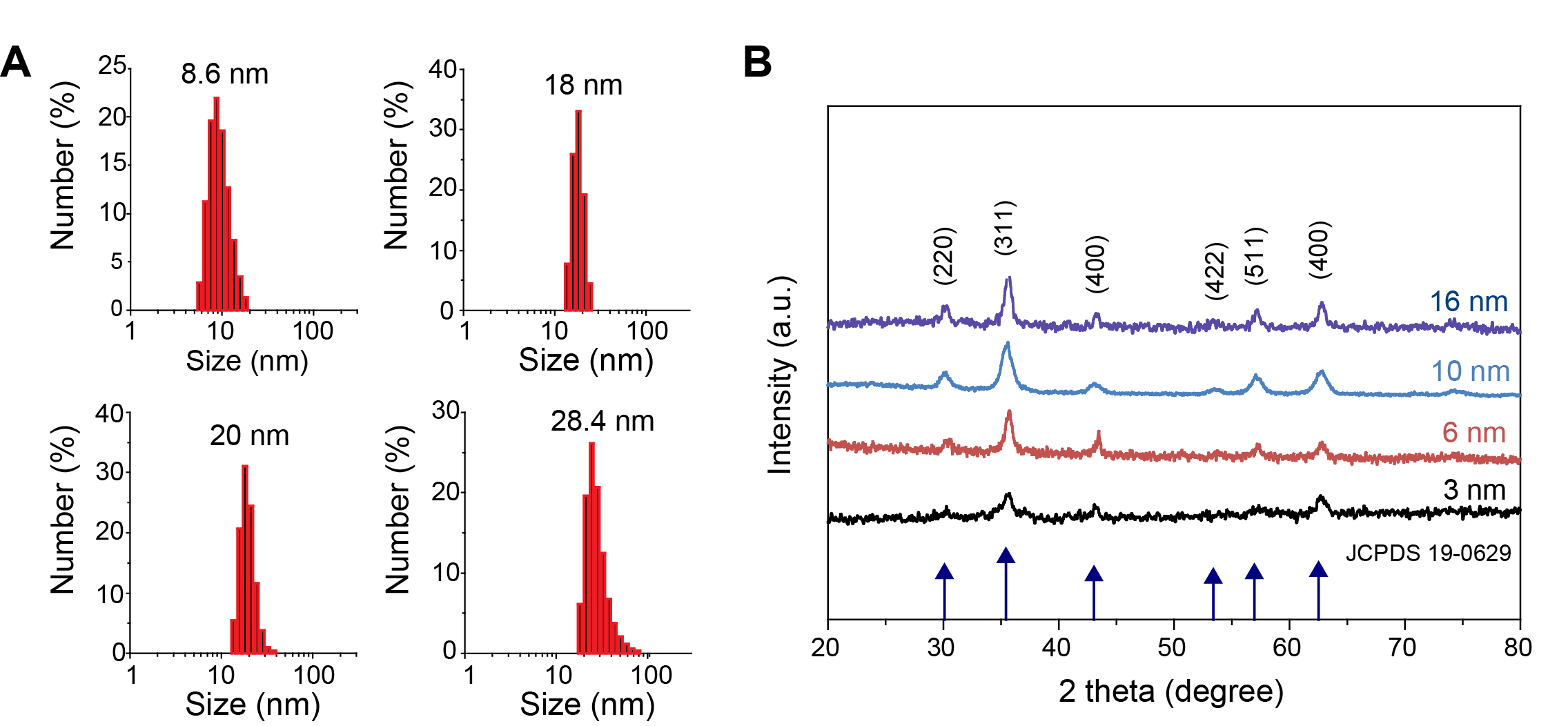


**Figure S1.** **Characterization of iron oxide nanoparticles (IONPs).** (**A**) Size distribution of 3 nm IONPs (IONP3), 6 nm IONPs (IONP6), 10 nm IONPs (IONP10) and 16 nm IONPs (IONP16). (**B**) XRD pattern of IONPs (IONP3-black, IONP6-red, IONP10-blue, IONP16-purple).


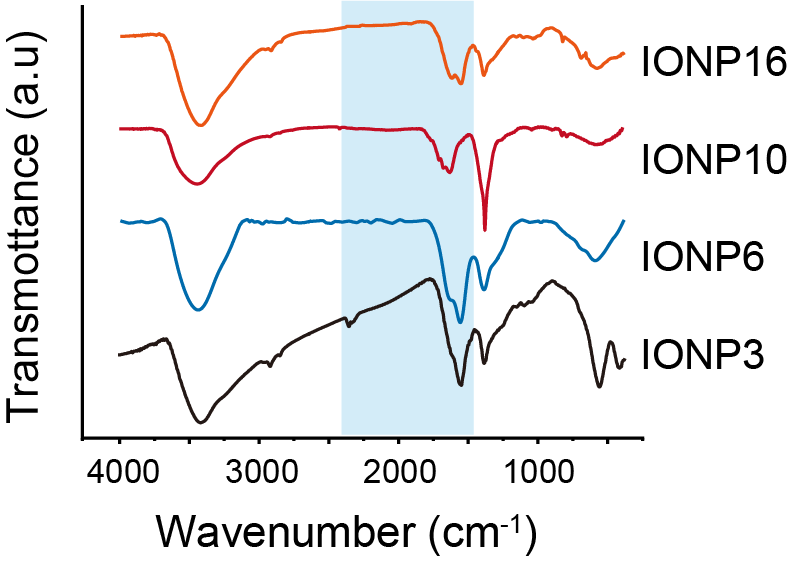


**Figure S2.** **Fourier transform infrared spectroscopy of IONP3, IONP6, IONP10 and IONP16.**


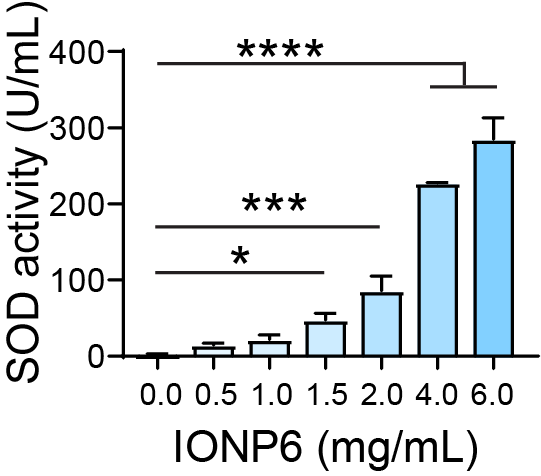


**Figure S3.** **The SOD activity of IONP6 at different concentrations.** n = 3 independent experiments. The data are presented as the mean ± SEM. P-values are calculated by one-way analysis of variance (ANOVA), **p* < 0.05, ****p* < 0.001, *****p* < 0.0001, 0.5, 1.0, 1.5, 2.0, 4.0 or 6.0 mg/mL IONP6 vs. the 0.0 mg/mL IONP6 group.


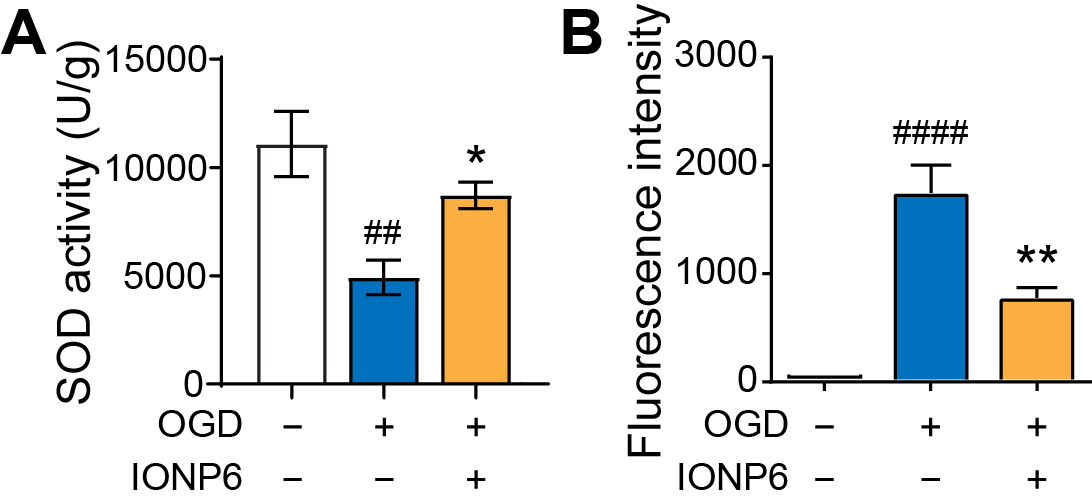


**Figure S4. SOD activities and RONS scavenging of the IONP6.** (**A**) The SOD activity of OGD-induced BV-2 cells with or without treatment with 25 μg/mL IONP6 (n = 3 independent experiments). (**B**) Quantification of cellular ROS levels with or without IONP6 treatment (n = 5 technical replicates from 3 independent experiments). BV-2 cells incubated with fresh medium without OGD induced served as the control group. BV-2 cells with OGD induced served as the OGD group. BV-2 cells with OGD induced and treated with IONP6 served as the IONP6 group. The data are presented as mean ± SEM. P-values are calculated by one-way ANOVA. ^###^*p* < 0.001, LPS group vs the control group, ****p* < 0.001, *****p* < 0.0001, IONPs group vs. the LPS group. ^##^*p* < 0.01, ^####^*p* < 0.0001, OGD group vs. the control group, **p* < 0.05, ***p* < 0.01, IONP6 group vs. the OGD group.

**
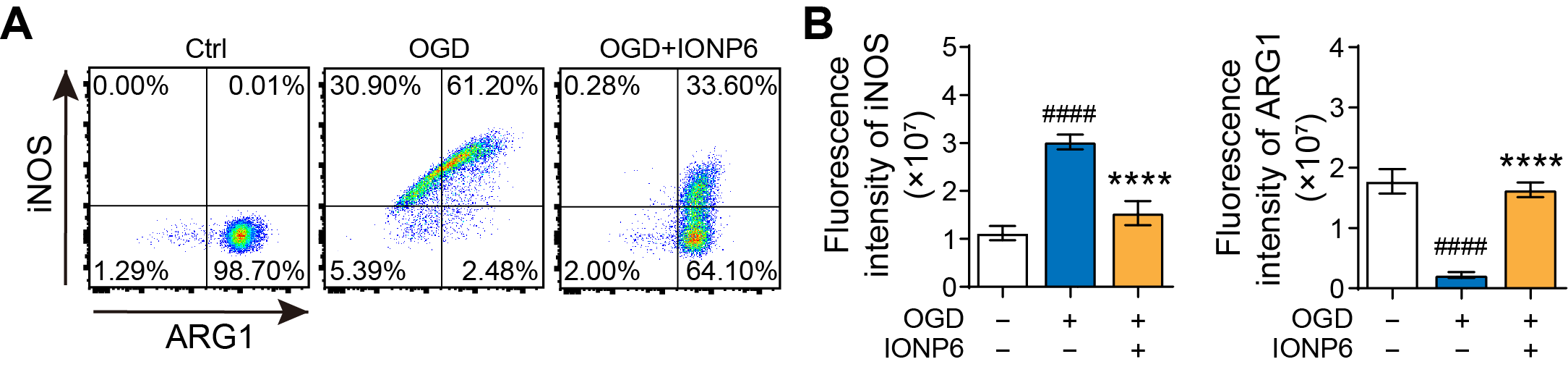
**

**Figure S5. The anti-neuroinflammatory effects of IONP6 *in vitro*.** (**A**) The cells were analyzed by flow cytometry using antibodies against iNOS and ARG1. Representative flow cytometry plots depicting the identification of iNOS and ARG1 in OGD-induced BV-2 cells with or without treatment with 25 μg/mL IONP6 (n = 3 biological replicates). (**B**) Mean fluorescence intensity of iNOS (left) and ARG1 (right) from the experiments shown in Figure 2I (n = 9 technical replicates from 3 independent experiments). OGD-induced BV-2 cells were treated with 25 μg/mL IONP6 or PBS for 6  h. The data are presented as the mean ± SEM. P-values are calculated by one-way ANOVA. BV-2 cells incubated with fresh medium without OGD induced served as the control group. BV-2 cells with OGD induced served as the OGD group. BV-2 cells with OGD induced and treated with IONP6 served as the IONP6 group. ^####^*p* < 0.0001, OGD group vs. the control group, *****p* < 0.0001, IONP6 group vs. the OGD group.


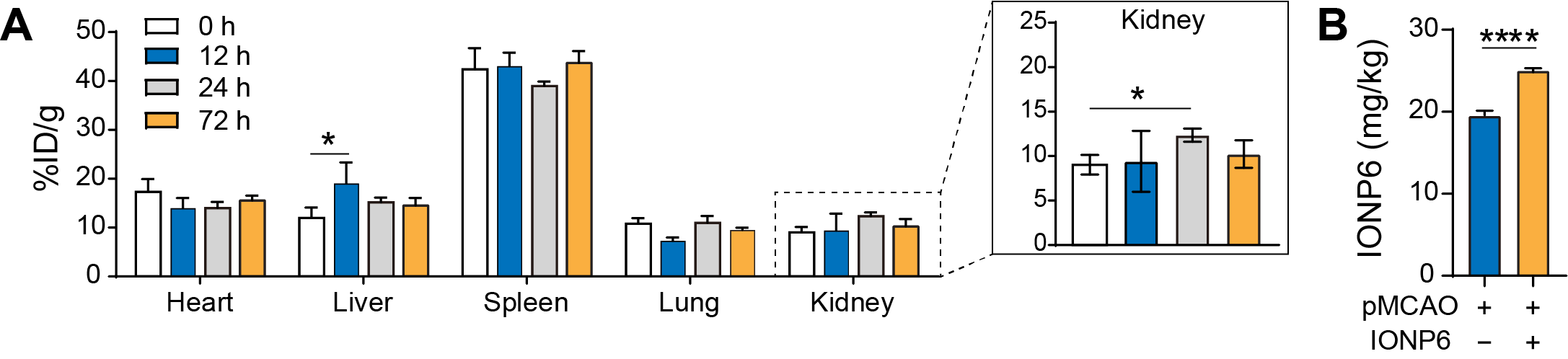


**Figure S6. The distribution of IONP6 *in vivo*.** (**A**) IONP6 distribution in the major organs at different time points. The right panel is a magnified view of the IONP6 distribution in the kidney. The organs of pMCAO rats (n = 5 rats per time point), intravenously injected with 5 mg/kg IONP6, were collected at the indicated times and analysed using Inductively Coupled Plasma Optical Emission Spectrometer (ICP-OES). The concentration of IONP6 was normalized as the percentage of the injected dose (ID) per gram of each organ (% ID/g). The data are presented as the mean ± SEM. P-values are calculated by two-way ANOVA in the left panel and unpaired t-test in the right panel. (**B**) Distribution of IONP6 in the ischemic hemisphere at 24 h. n = technical replicates from 3 rats per group. The data are presented as the mean ± SEM. P-values are calculated by unpaired t-test. **p* < 0.05, 12 h vs. 0 h group in liver, 24 h vs. 0 h group in kidney, *****p* < 0.0001, pMCAO + IONP6 group vs. the pMCAO group.


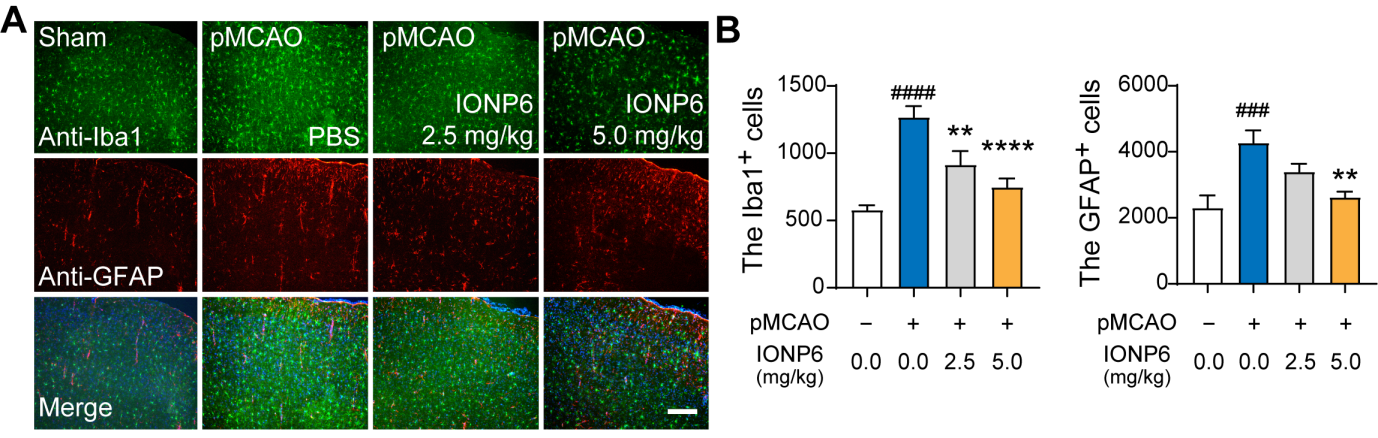


**Figure S7.** **IONP6 inhibited microglial and astrocyte activation.** (**A**) Immunofluorescent staining of microglia (anti-Iba1 labeled, green), astrocytes (anti-GFAP labeled, red) in the cortex of brains from sham rats, pMCAO rats with or without IONP6 treatment. Scale bar, 50 μm. (**B**) Quantitation of Iba1^+^ cells (left) and GFAP^+^ cells (right) (n = 8 technical replicates from 3 rats for 2.5 mg/kg IONP6 group, n = 9 technical replicates from 3 rats for other groups). (A and B) demonstrated that IONP6 reduced the activation of microglia and astrocytes. The data are presented as the mean ± SEM. P-values are calculated by one-way ANOVA. Rats without IONP6 treatment served as the sham group, pMCAO rats without IONP6 treatment served as the pMCAO group, and pMCAO rats with IONP6 treatment served as the IONP6 group. ^##^*p* < 0.01, ^###^*p* < 0.001, ^####^*p* < 0.0001, pMCAO group vs. the sham group, **p* < 0.05, ***p* < 0.01, *****p* < 0.0001, 2.5 mg/kg or 5 mg/kg IONP6 group vs. the pMCAO group.


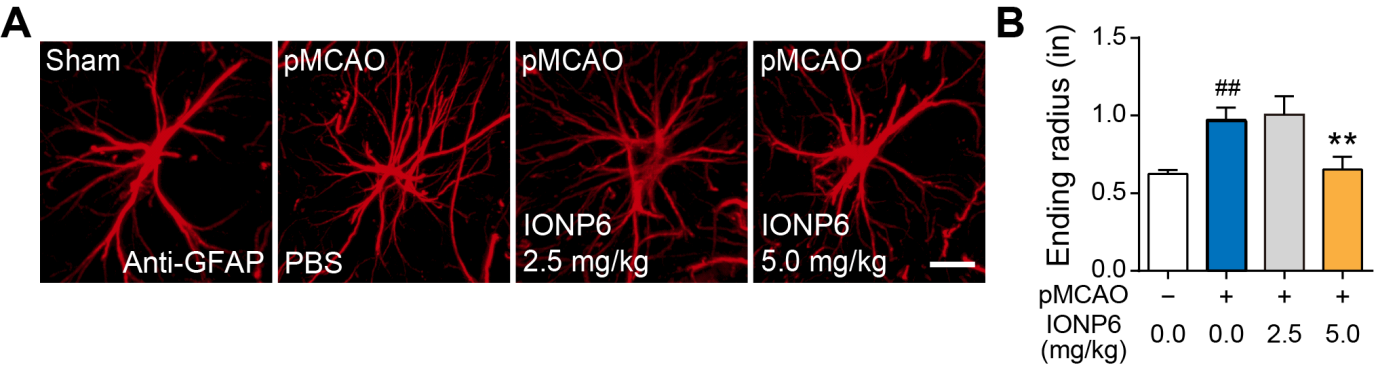


**Figure S8.** **IONP6 inhibited astrocyte activation.** (**A**) The morphology of astrocytes was observed by GFAP-staining in the hippocampus of brains from sham rats, pMCAO rats with or without IONP6 treatment. Scale bar, 20 μm. (**B**) Quantitative analysis of the ending radius by Sholl analysis in panel A (n = 3 biological replicates). The data are presented as the mean ± SEM. P-values are calculated by one-way ANOVA. ^##^*p* < 0.01, pMCAO group vs. the sham group, ***p* < 0.01, 2.5 mg/kg or 5 mg/kg IONP6 group vs. the pMCAO group.


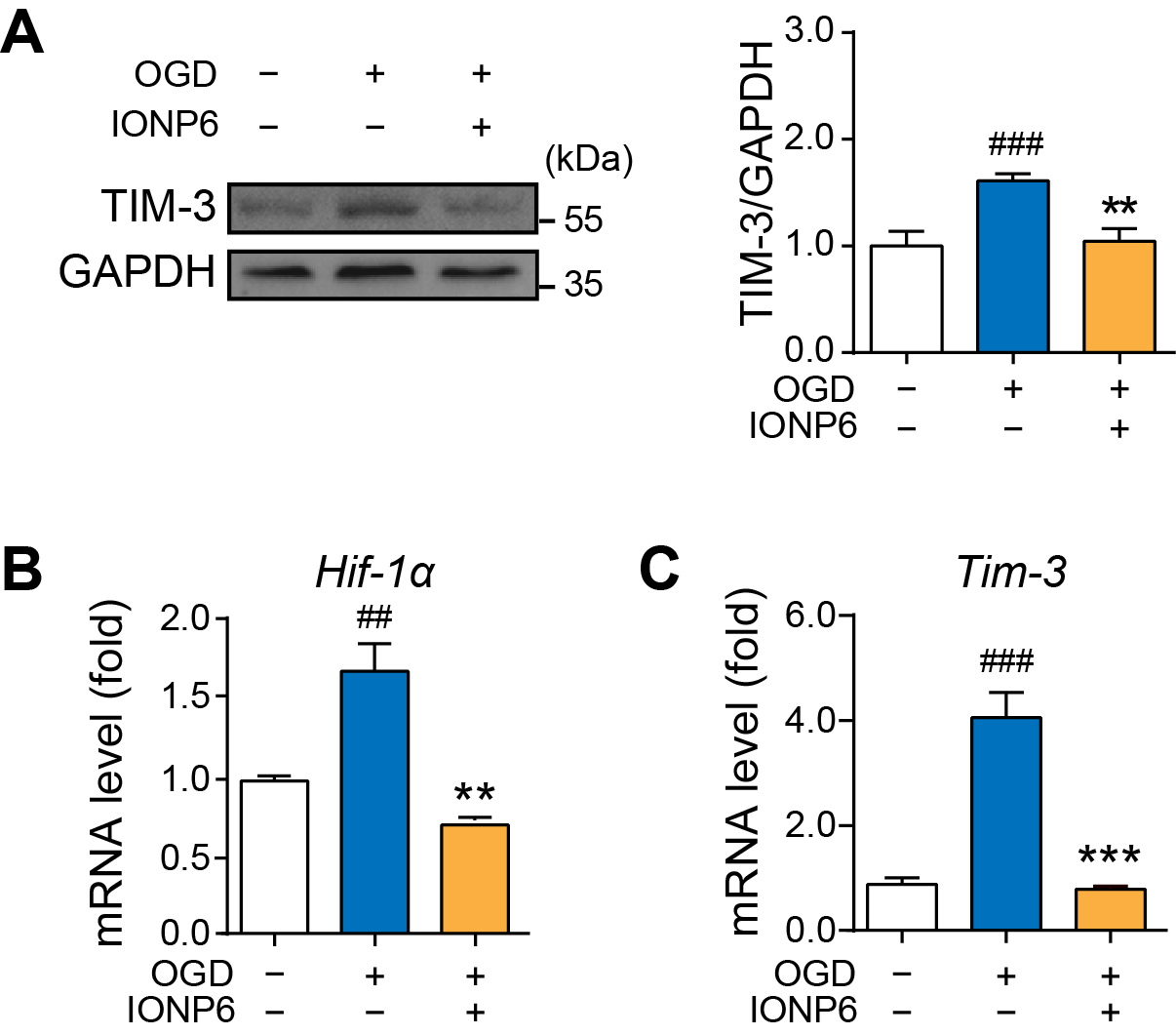


**Figure S9.** **IONP6 attenuated inflammatory responses via the HIF-1α/TIM-3 axis *in vitro*.** (**A**) Western blot analysis of TIM-3 protein expression in OGD-induced BV-2 cells, with or without treatment with 25 μg/mL IONP6 (n = 3 biological replicates). (**B** and **C**) The mRNA expression levels of *Hif-1α* (B) and *Tim-3* (C) were detected by RT-qPCR in BV-2 cells (n = 3 biological replicates). The data are presented as the mean ± SEM. P-values are calculated by one-way ANOVA. BV-2 cells incubated with fresh medium without OGD induced served as the control group. BV-2 cells with OGD induced served as the OGD group. BV-2 cells with OGD induced and treated with IONP6 served as the IONP6 group. ^#^*p* < 0.05, ^###^*p* < 0.001, ^####^*p* < 0.0001, OGD group vs. the control group, **p* < 0.05, ***p* < 0.01, ****p* < 0.001, *****p* < 0.0001, IONP6 group vs. the OGD group.


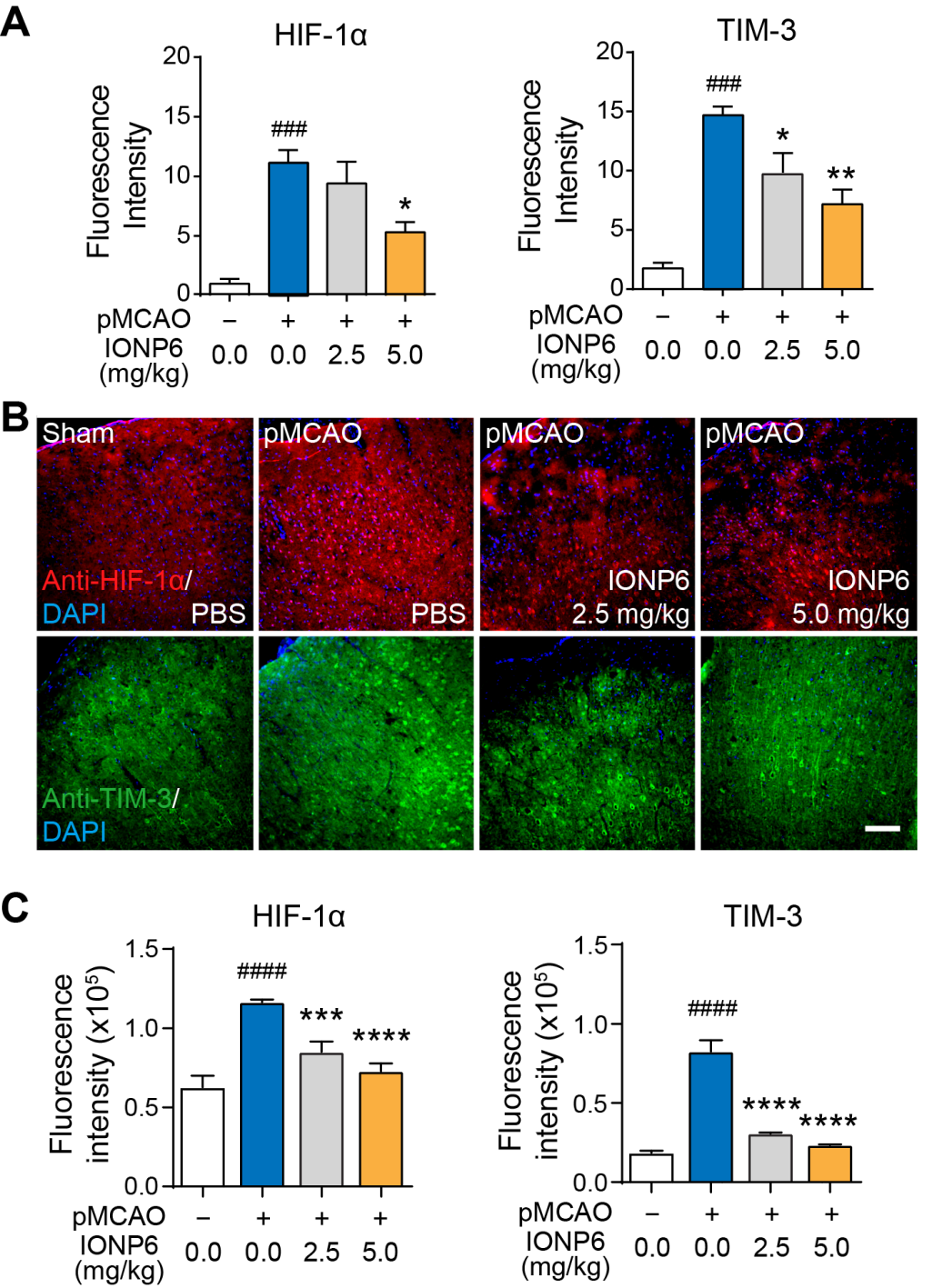


**Figure S10.** **IONP6 attenuated inflammatory responses via the HIF-1α/TIM-3 axis *in vivo*.** (**A**) Quantitative analysis of different groups in Figure 4H. n = 3. (**B**) Immunohistochemistry was performed in the cortical regions of brains from sham rats and pMCAO rats, with or without IONP6 treatment, using anti-HIF-1α (red) and anti-TIM-3 (green) antibodies. Scale bar, 25 μm. (**C**) Quantitative analysis of different groups in panel B. Rats without IONP6 treatment served as the sham group, pMCAO rats without IONP6 treatment served as the pMCAO group, and pMCAO rats with IONP6 treatment served as the IONP6 group. n = 12 independent slices from 3 rats for the 2.5 mg/kg IONP6 group, n = 13 independent slices from 3 rats for other groups in the left panel. n = 13 independent slices from 3 rats for all groups in the right panel. The data are presented as the mean ± SEM. P-values are calculated by one-way ANOVA. ^###^*p* < 0.001, ^####^*p* < 0.0001, pMCAO group vs. the sham group, **p* < 0.05, ***p* < 0.01, ****p* < 0.001, *****p* < 0.0001, 2.5 mg/kg or 5 mg/kg IONP6 group vs. the pMCAO group.

**
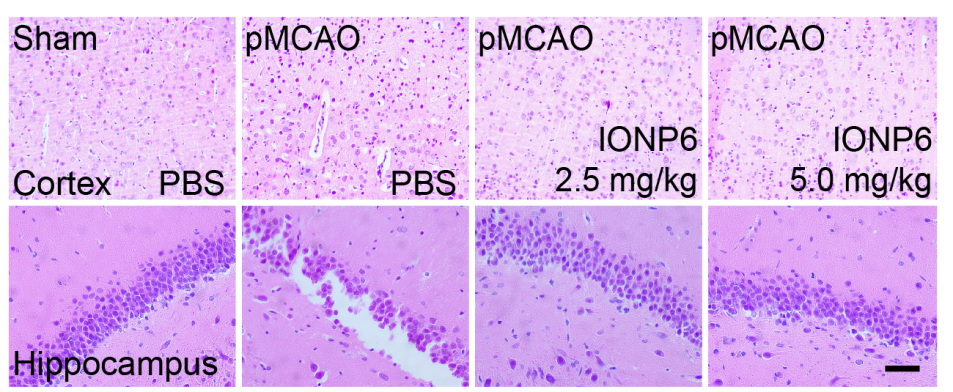
**

**Figure S11.** **Detection of neuronal apoptosis by hematoxylin and eosin (H&E) staining.** Representative images for H&E staining in the cortex and hippocampus of brains from sham rats, pMCAO rats with or without IONP6 treatment. Scale bar, 12.5 μm.


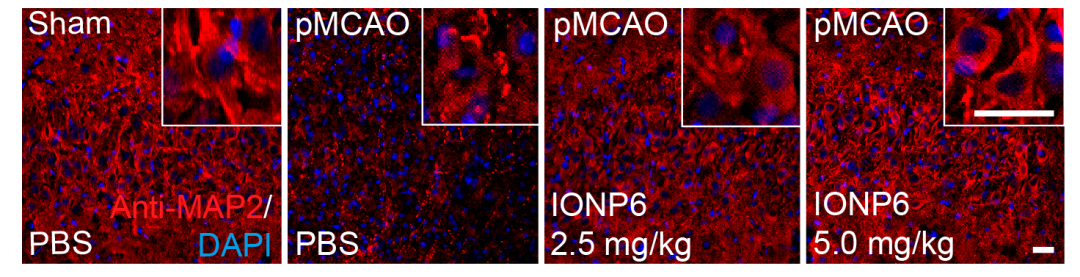


**Figure S12.** **Protective effect of IONP6 on neurons.** Representative images of neurons (anti-MAP2 labeled, red) in the cortex of brain sections from sham rats, pMCAO rats with or without IONP6 treatment. Scale bar, 25 μm.


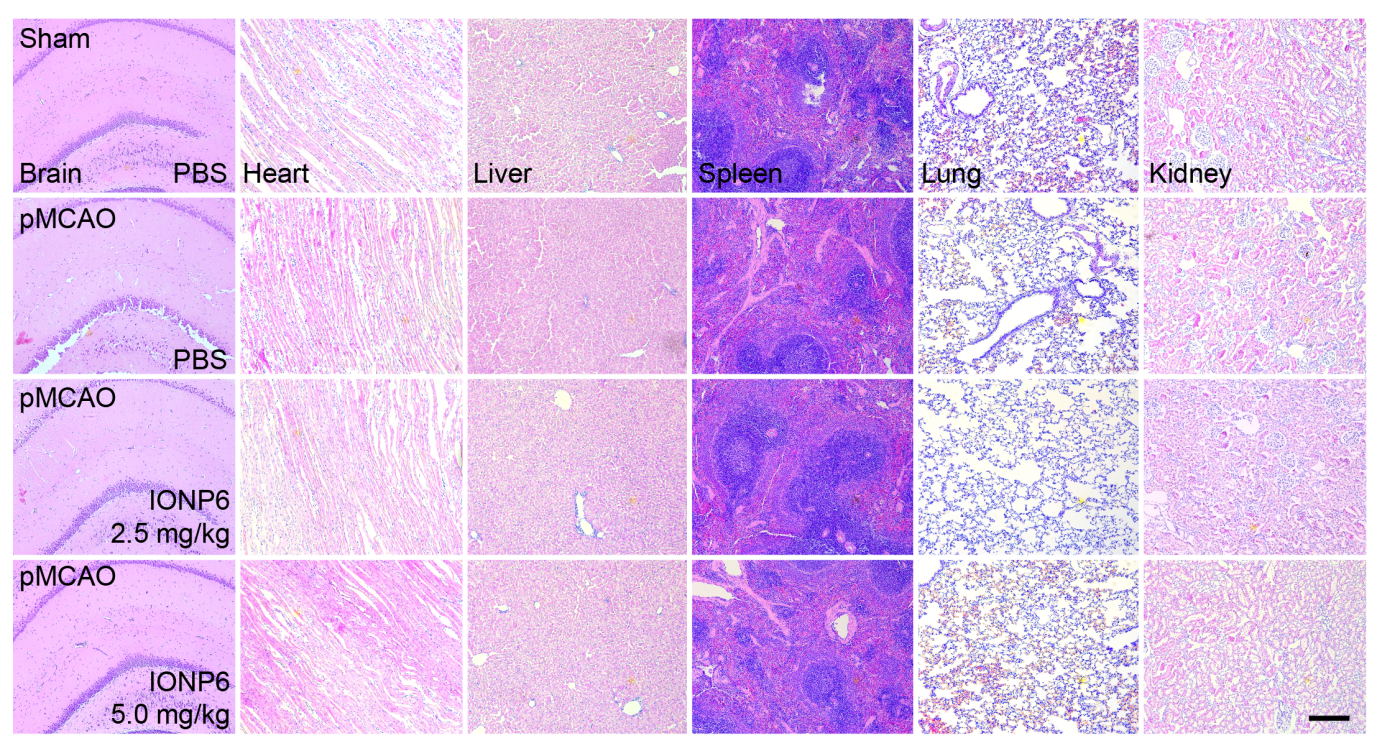


**Figure S13.** **Representative H&E-stained sections of major organs from sham rats, pMCAO rats with or without IONP6 treatment.** Histology performed on organs after IONP6 treatment, which exhibited imperceptible organ damage, but had a protective effect on the brain tissue of stroke rats. Scale bar, 50 μm.

**Table S1.** Fourier transform infrared spectroscopy of reaction products of IONPs and NO.

| Wavenumbers（cm^-1^） | Assignment |
| --- | --- |
| 2930 | -CH2- |
| 2850 | -CH2- |
| 2168 | N-O |
| 1790 | Fe-N |
| 1712 | N-O |
| 1561 | COOH |
| 1385 | -CH3 |
| 831 | C-H |
| 539 | Fe-O |

**Table S2.** XPS analysis of reaction products of IONPs and NO showing the binding energy (BE) levels of N1s.

| IONPs size | Location (ev) | Assignment | Area |
| --- | --- | --- | --- |
| 3 nm | 399.84  405.01  406.88  400.33  405.2  406.8  400.02  406.3  400.12  406.2 | Fe-N  NO  $\text{NO}_{\text{3}}^{\text{-}}$  Fe-N  NO  $\text{NO}_{\text{3}}^{\text{-}}$  Fe-N  $\text{NO}_{\text{3}}^{\text{-}}$  Fe-N  $\text{NO}_{\text{3}}^{\text{-}}$ | 2225.615  3204.675  539.513  4334.618  2152.899  1933.535  3901.239  2310.374  2299.443  2137.212 |
| 6 nm |  |  |  |
| 10 nm |  |  |  |
| 16 nm |  |  |  |

**Table S3.** Primer sequences.

| ID | Primer sequence |
| --- | --- |
| *β-Actin*  *m-Il-6*  *m-Il-1β*  *m-Tnf-α*  *m-Il-4*  *m-Il-10*  *m-Arg-1*  *m-Hif-1α*  *m-Tim-3*  *r-Il-6*  *r-Tnf-α*  *r-Arg1*  *r-Il-1β* | Forward:5’-GGCTGTATTCCCCTCCATCG-3’  Reverse:5’-CCAGTTGGTAACAATGCCATGT-3’  Forward:5’-AGCCAGAGTCCTTCAGAGAG-3’  Reverse:5’-CTTAGCCACTCCTTCTGTGAC-3’  Forward:5’-TGTGTAATGAAAGACGGCAC-3’  Reverse:5’-TCCACTTTGCTCTTGACTTC-3’  Forward:5’-CAAAATTCGAGTGACAAGCCT-3’  Reverse:5’-CTGGGAGTAGACAAGGTACAAC-3’  Forward:5’-GAGTGAGCTCGTCTGTAGGG-3  Reverse:5’-GAAGTCTTTCAGTGATGTGG-3  Forward:5’-CCAACCTGAGCATCTTAGTC-3  Reverse:5’-TGGGAAAACCTCCAGGTCCA-3  Forward:5’-CCAACCTGAGCATCTTAGTC-3  Reverse:5’-TGGGAAAACCTCCAGGTCCA-3  Forward:5’-CTCATCAGTTGCCACTTCC-3  Reverse:5’-TCATCTTCACTGTCTAGACCAC-3  Forward:5’-CCCTGCAGTTACACTCTACC-3  Reverse:5’-GTATCCTGCAGCAGTAGGTC-3  Forward:5’-GCCTATTGAAAATCTGCTCTGG-3  Reverse:5’-GGAAGTTGGGGTAGGAAGGA-3  Forward:5’-GCATGATCCGAGATGTGGAACTG-3  Reverse:5’-CGCCACGAGCAGGAATGAGAAG-3  Forward:5’-TGCCGTGTTCACAGTACGAGTC-3  Reverse:5’-AAGGAAGAAAAGGCCCATTCA-3  Forward:5’-GGATGAGGACATGAGCACCT-3  Reverse:5’-TCCATTGAGGTGGAGAGCTT-3 |
